# IRG and GBP Host Resistance Factors Target Aberrant, “Non-self” Vacuoles Characterized by the Missing of “Self” IRGM Proteins

**DOI:** 10.1371/journal.ppat.1003414

**Published:** 2013-06-13

**Authors:** Arun K. Haldar, Hector A. Saka, Anthony S. Piro, Joe Dan Dunn, Stanley C. Henry, Gregory A. Taylor, Eva M. Frickel, Raphael H. Valdivia, Jörn Coers

**Affiliations:** 1 Departments of Molecular Genetics and Microbiology, and Immunology, Duke University Medical Center, Durham, North Carolina, United States of America; 2 Department of Molecular Genetics and Microbiology, Duke University Medical Center, Durham, North Carolina, United States of America; 3 Departments of Medicine, Molecular Genetics and Microbiology, and Immunology and Center for the Study of Aging, Duke University, Durham, North Carolina, United States of America; 4 Geriatric Research and Education and Clinical Center, Veteran Affairs Medical Center, Durham, North Carolina, United States of America; 5 Division of Parasitology, MRC National Institute for Medical Research, London, United Kingdom; Stanford University School of Medicine, United States of America

## Abstract

Interferon-inducible GTPases of the Immunity Related GTPase (IRG) and Guanylate Binding Protein (GBP) families provide resistance to intracellular pathogenic microbes. IRGs and GBPs stably associate with pathogen-containing vacuoles (PVs) and elicit immune pathways directed at the targeted vacuoles. Targeting of Interferon-inducible GTPases to PVs requires the formation of higher-order protein oligomers, a process negatively regulated by a subclass of IRG proteins called IRGMs. We found that the paralogous IRGM proteins Irgm1 and Irgm3 fail to robustly associate with “non-self” PVs containing either the bacterial pathogen *Chlamydia trachomatis* or the protozoan pathogen *Toxoplasma gondii*. Instead, Irgm1 and Irgm3 reside on “self” organelles including lipid droplets (LDs). Whereas IRGM-positive LDs are guarded against the stable association with other IRGs and GBPs, we demonstrate that IRGM-stripped LDs become high affinity binding substrates for IRG and GBP proteins. These data reveal that intracellular immune recognition of organelle-like structures by IRG and GBP proteins is partly dictated by the *missing of “self”* IRGM proteins from these structures.

## Author Summary

Cell-autonomous host defense pathways directed against vacuolar pathogens constitute an essential arm of the mammalian innate immune defense system. Underlying most of these defense strategies is the ability of the host cell to recognize foreign or pathogen-modified structures and to deliver antimicrobial molecules specifically to these sites. Specific targeting of molecules to pathogen-containing vacuoles (PVs) requires host cells to recognize PVs as “non-self” structures that are distinct from intact “self” structures like organelles and other endomembrane components. In this work, we develop a new framework for understanding a critical principle that guides the mammalian immune system in the recognition of PVs as “non-self” structures. Our data indicates that so-called IRGM proteins function as markers of “self” compartments. We find that IRGM proteins act as “guards” that prevent a set of antimicrobial GTPases from stable association with “self” membranes. Because IRGM proteins are largely absent from “non-self” PVs, we propose that intracellular immune recognition of PVs can occur via the missing of “self” IRGM proteins.

## Introduction

Many intracellular pathogens including the bacterium *C. trachomatis* and the protozoa *T. gondii* co-opt the host cell endomembrane system to enclose themselves inside membrane-bound vacuoles. Within the confines of these remodeled PVs, microbes acquire nutrients and replicate [1]. To combat these pathogens, the mammalian host has evolved a large repertoire of cell-autonomous defense mechanisms that kill or restrain the replication of microbes residing within vacuoles [2], [3]. While these defense mechanisms are effective at targeting foreign or “non-self” vacuoles, they also have the potential to cause organelle damage and must therefore be tightly regulated. Control over these host defenses is executed at two critical steps: (i) induction of genes encoding host resistance factors in the context of an infection and (ii) targeting of these resistance factors to the appropriate intracellular location, for example to PVs.

These two modes of regulation are exemplified by the induction and execution of cell-autonomous defenses by the cytokine Interferon-γ (IFNγ). The importance of IFNγ in host immunity is demonstrated by the severe immuno-deficiencies observed in genetically engineered mouse strains lacking IFNγ or its receptor and in patients carrying rare mutations in genes critical for IFNγ signal transduction [4], [5]. IFNγ is produced by immune-activated lymphocytes and exerts its antimicrobial effects by dramatically remodeling the transcriptional expression profile of target cells bearing the IFNγ receptor [3]. IFNγ-induced resistance genes include members of two IFN-inducible GTPase families named IRGs and GBPs. Members of both GTPase families have the ability to translocate and to adhere specifically to PVs in order to inhibit intracellular pathogen growth. Although the specificity of this intracellular targeting event is well documented [3], [6], the underlying mechanism is unclear.

Once docked to PVs, GBP proteins recruit antimicrobial protein complexes that include the NADPH oxidase NOX2, the autophagy apparatus and the inflammasome [3]. IRG proteins on the other hand can directly disrupt PV membranes, thereby releasing vacuolar pathogens into the cytosol where they can be removed through autophagy [6], [7]. IRG GTPases are divided into two categories: (i) the predominantly cytosolic GKS proteins constitute the most abundant group and harbor a conserved GX_4_ **G**K**S** sequence in the first nucleotide-binding motif (G1), (ii) the predominantly membrane-bound IRGM proteins instead contain a non-canonical P-loop sequence GX_4_ **G**M**S** [6]. Both GKS and IRGM proteins are essential for cell-autonomous resistance to infections with *C. trachomatis* and *T. gondii* in mice but fulfill distinct functions in this process [6]. Whereas GKS proteins directly target and eliminate *C. trachomatis* and *T. gondii* PVs, IRGM proteins appear to orchestrate the targeting of GKS proteins to PVs by an incompletely understood mechanism [6]. In addition to their role as regulators of GKS protein function, IRGM proteins also exert antimicrobial activities independently of GKS proteins. Both mouse and human IRGM proteins promote the formation of autophagosomes upon IFNγ stimulation [8]–[10]. Additionally, murine Irgm1 loads onto early phagosomes containing beads or live bacteria [11]–[13]. Vacuolar Irgm1 interacts with target SNARE protein complexes and through these interactions can facilitate the rapid fusion of Irgm1-coated phagosomes with degradative lysosomes. Accelerated lysosomal maturation was shown to result in the destruction of the attenuated pathogen *Mycobacterium bovis* BCG contained within Irgm1-positive phagosomes in mouse macrophages [13]. Similar to Irgm1, Irgm3 was implicated as a mediator of direct antimicrobial activities towards *T. gondii* [14].

To initially establish vacuoles permissive for microbial survival in IFNγ-activated cells, pathogens must have evolved strategies to evade the direct, fast-acting immune responses mediated by membrane-bound Irgm1 and Irgm3 proteins. In agreement with the existence of such evasion mechanisms, we observed that Irgm1 and Irgm3 failed to robustly associate with PVs formed by either *C. trachomatis* or *T. gondii*. The absence of substantial amounts of Irgm1/m3 from PVs contrasted with the abundant localization of Irgm1/m3 to “self” structures like LDs. We found that Irgm1/m3-decorated LDs are largely devoid of GKS and GBP proteins, whereas Irgm1/m3-deficient PVs are targets for GKS and GBP proteins. These observations led us to hypothesize that the absence of Irgm1/m3 proteins marked intracellular structures as targets for a “second line of defense” mediated by GKS and GBP proteins. In support of this hypothesis, we demonstrated that stripping LDs of Irgm1/3 resulted in mistargeting of GKS and GBP proteins to LDs independently of an infection. Because IRGM proteins were previously shown to inhibit GKS protein oligomerization [15], we propose a model in which the *missing of “self”* IRGM proteins from “non-self” PVs results in the formation of GKS (and GBP) protein oligomers with high avidity for membrane binding.

## Results

### Protein oligomerization is necessary and sufficient to target Irgb10 to *C. trachomatis* inclusions

**Figure 1:**
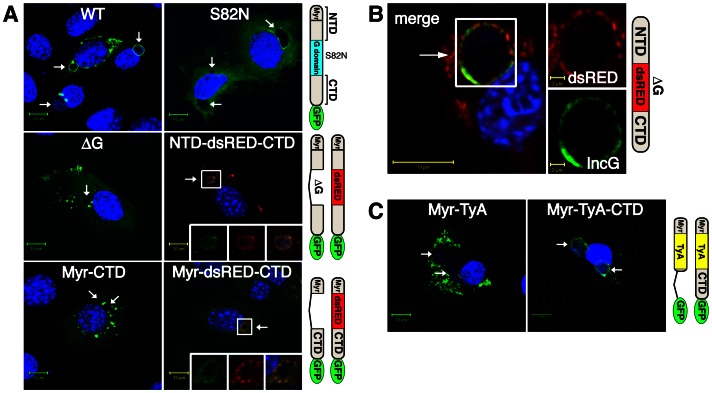
Oligomerization of the N- and C-terminus of Irgb10 is sufficient to target GFP-fusion proteins to *C.* *trachomatis* inclusions. (**A and C**) The indicated GFP fusion proteins were ectopically expressed in wildtype MEFs. Transfected cells were then infected with *C. trachomatis* and treated with IFNγ at 3 hpi. Cells were fixed and stained for DNA with Hoechst at 20 hpi. Arrows point at Hoechst-positive inclusions. (**B**) Cells were transfected with NTD-dsRED-CTD lacking a GFP moitety, infected with *C. trachomatis* and stained with anti-IncG.

GKS proteins in their GTP-bound state form dimers [6]. Dimerization occurs at the G domain interface and is a prerequisite for the formation of higher order GKS protein oligomers. Mutations that diminish guanine nucleotide binding or disrupt the G domain interface eliminate both protein oligomerization and targeting to *T. gondii* PVs [16], [17]. To determine if these findings extended to other PVs, we first tested whether guanine nucleotide binding of the GKS protein Irgb10 was essential for their targeting to “inclusions,” the PVs formed by *C. trachomatis*. We replaced the serine on position 82 of Irgb10 in the conserved P-loop GKS motif with asparagine (Irgb10^S82N^). This mutation is analogous to the Irga6^S83N^ mutation that abrogates guanine nucleotide binding and *T. gondii* PV localization [16]. We found that Irgb10-GFP-fusion proteins harboring the S82N mutation or a deletion of the central G-domain (Irgb10ΔG) failed to localize to *C. trachomatis* inclusions in infected mouse embryonic fibroblasts (MEFs) (Figure 1A).

**Figure 2:**
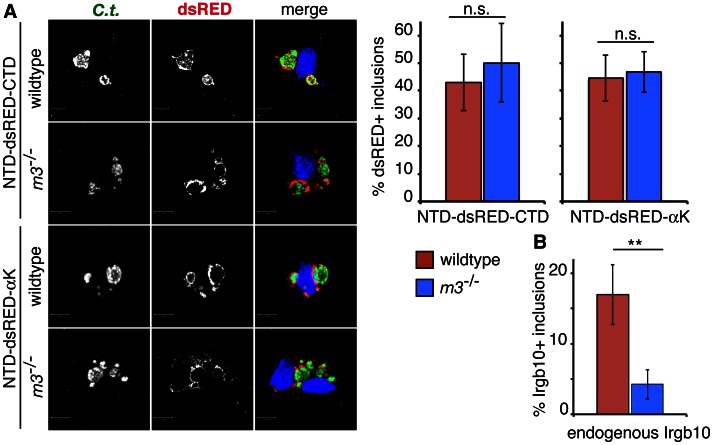
Tetramerized Irgb10 targets inclusions independently of Irgm3. (**A**) Wildtype and *Irgm3* ^−/−^ MEFs were transfected with Irgb10^NTD-dsRED-CTD^ or Irgb10^NTD-dsRED-αK^, subsequently infected with *C. trachomatis* and treated with IFNγ at 3 hpi. Fixed cells were stained with anti-*C. trachomatis* and Hoechst. The frequency at which dsRED fusions proteins co-locliazed with incluions inside transfected cells at 20 hpi was quantified in three independent experiments. Representative data from one experiment and images at 20 hpi are shown. Graph represents average values ± SD. (**B**) Wildtype MEFs were infected with *C. trachomatis*, treated with IFNγ at 3 hpi and the frequency of endogenous Irgb10 localizing to inclusions at 20 hpi was quantified. Error bars represent standard deviations. n.s. = not significant; **, p<0.01.

Combined with previous results in *T. gondii* [16], our data suggested that protein oligomerization of GTP-bound Irgb10 is essential for tethering this GKS protein to *C. trachomatis* inclusion membranes. To test whether protein oligomerization of the N- and C-terminal domains of Irgb10 was sufficient to target inclusion membranes, we replaced the G domain of Irgb10 with alternative protein oligomerization domains and monitored the subcellular localization of these protein chimeras. We first substituted the G domain of Irgb10 with the tetramer-forming protein dsRED [18], which emits red fluorescence exclusively in the oligomerized form [19]. Insertion of dsRED between the N-terminal domain (NTD) and C-terminal domain (CTD) of Irgb10ΔG (Irgb10^NTD-dsRED-CTD^) restored the association of a GFP-tagged fusion protein with *C. trachomatis* inclusions (Figure 1A). Inserting dsRED in between the N-terminal myristoylation motif (Myr) and the CTD of Irgb10 (Irgb10^Myr-dsRED-CTD^) similarly redirected the mutant variant Irgb10^Myr-CTD^ to inclusions (Figure 1A). Tetramerized Irgb10 localized to IncG-positive inclusion membranes (Figure 1B). Inclusion targeting required the presence of both the Irgb10 myristoylation motif and the C-terminal amphipathic helix αK (Figure 1A, Figure 2A and data not shown). As an alternative mediator of protein oligomerization, we used the highly oligomeric cytoplasmic yeast protein TyA [20]. Insertion of TyA in between the myristoylation domain and the C-terminus of Irgb10 (Irgb10^Myr-TyA-CTD^) similarly re-localized these fusion proteins towards inclusions. Myristoylated-TyA (Myr-TyA) localized to microvesicles, as described [21], but failed to associate with inclusions (Figure 1C), demonstrating that the C-terminus of Irgb10 containing the αK amphipathic helix is essential for inclusion targeting. In summary, these data show that the oligomerization of the N- and C-terminal lipid binding domains of Irgb10 was sufficient to drive localization to inclusions.

### Tetramerized Irgb10 targets inclusions independently of Irgm3

**Figure 3:**
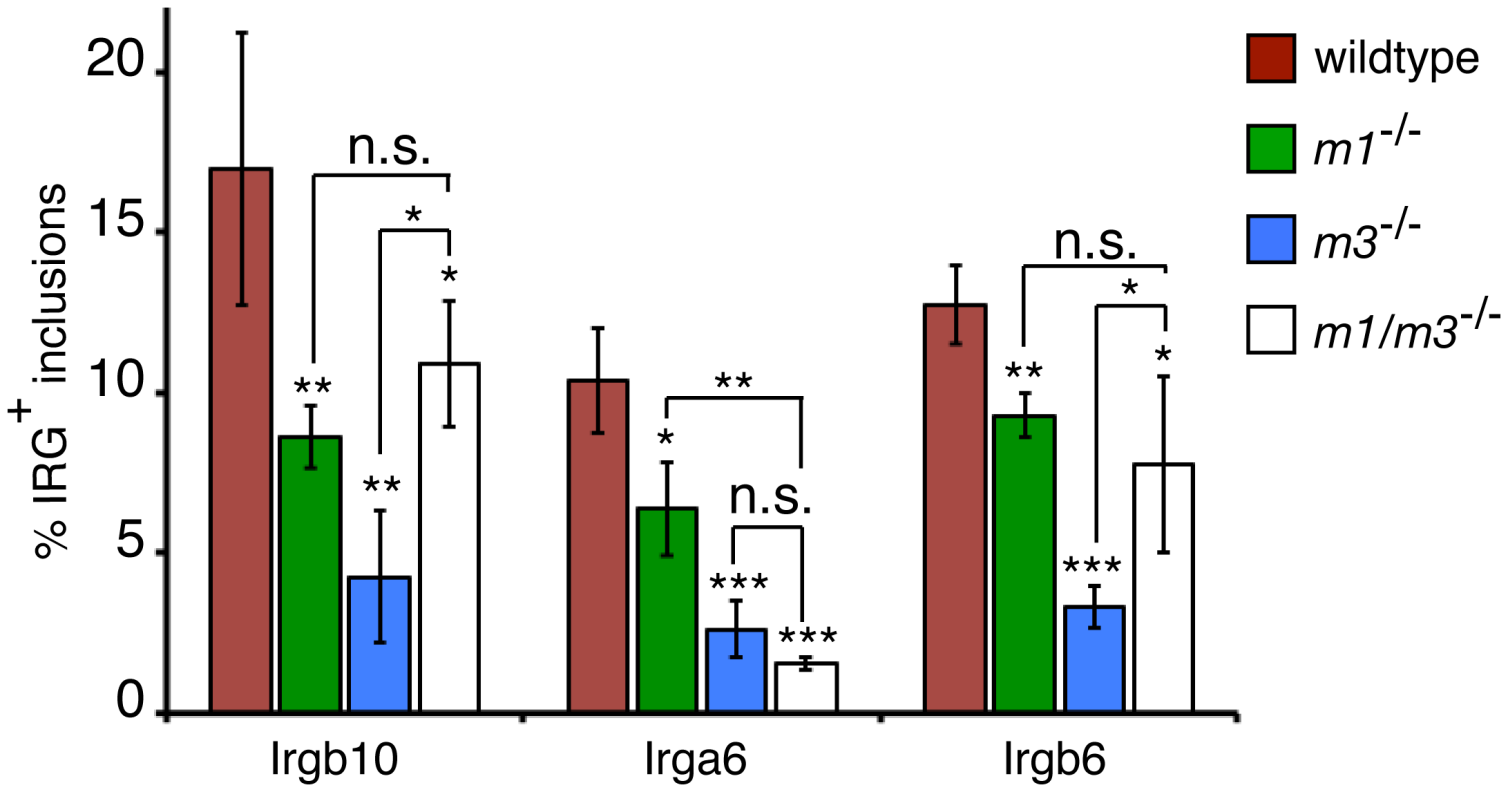
Irgm1 and Irgm3 are critical co-factors for targeting the GKS proteins Irgb10, Irga6 and Irgb6 to *C.* *trachomatis* inclusions. MEFs were infected with *C. trachomatis* and media +/− IFNγ at 100 U/ml was replaced at 3 hpi. Cells were fixed at 20 hpi and stained with anti-*C. trachomatis* and anti-GKS antibodies. The frequency of GKS-positive inclusions is shown in MEFs of the indicated genotypes. Graph represents average values ± SD. Data are representiaitve of three independent experiments. Statistical significance of group values relative to wildtype and between marked groups are shown (*, p<0.05; **, p<0.01; and ***, p<0.005).

The targeting of GKS proteins to *T. gondii* is substantially diminished in the absence of the IRGM proteins Irgm1 and Irgm3 [16], [22]. We found that the association of Irgb10 and other GKS proteins with *C. trachomatis* inclusions was similarly reduced in infected MEFs derived from *Irgm1* ^−/−^, *Irgm3* ^−/−^ and *Irgm1/m3^−/−^* mice (Figure 3). These data indicate that Irgm1 and Irgm3 either directly or indirectly promote the delivery of GKS proteins to inclusions. Because IRGM proteins physically interact with GKS proteins at the G domain interface [16], we hypothesized that IRGM proteins facilitate the delivery of GKS proteins to inclusions through their interactions with the G domain of GKS proteins. In such a scenario, artificially oligomerized Irgb10 lacking a G domain should target inclusions independently of IRGM proteins. To test the hypothesis, we expressed two tetramerized, chimeric Irgb10ΔG proteins, Irgb10^NTD-dsRED-CTD^ and Irgb10^NTD-dsRED-αK^, in wildtype and *Irgm3* ^−/−^ MEFs and scored the frequency of dsRED signal on inclusions. We chose *Irgm3* ^−/−^ MEFs for these experiments, because they displayed the most pronounced defect in targeting endogenous Irgb10 to inclusions (Figure 3). In contrast to endogenous Irgb10 (Figure 2B and Figure 3), tetramerized Irgb10 lacking a G domain targeted inclusions with the same efficiency in wildtype and *Irgm3*-deficient cells (Figure 2A). These data suggest that Irgm3 regulates the targeting of Irgb10 to inclusions through its interaction with the G domain of Irgb10.

### Irgm1 and Irgm3 strongly associate with LDs but not PVs

**Figure 4:**
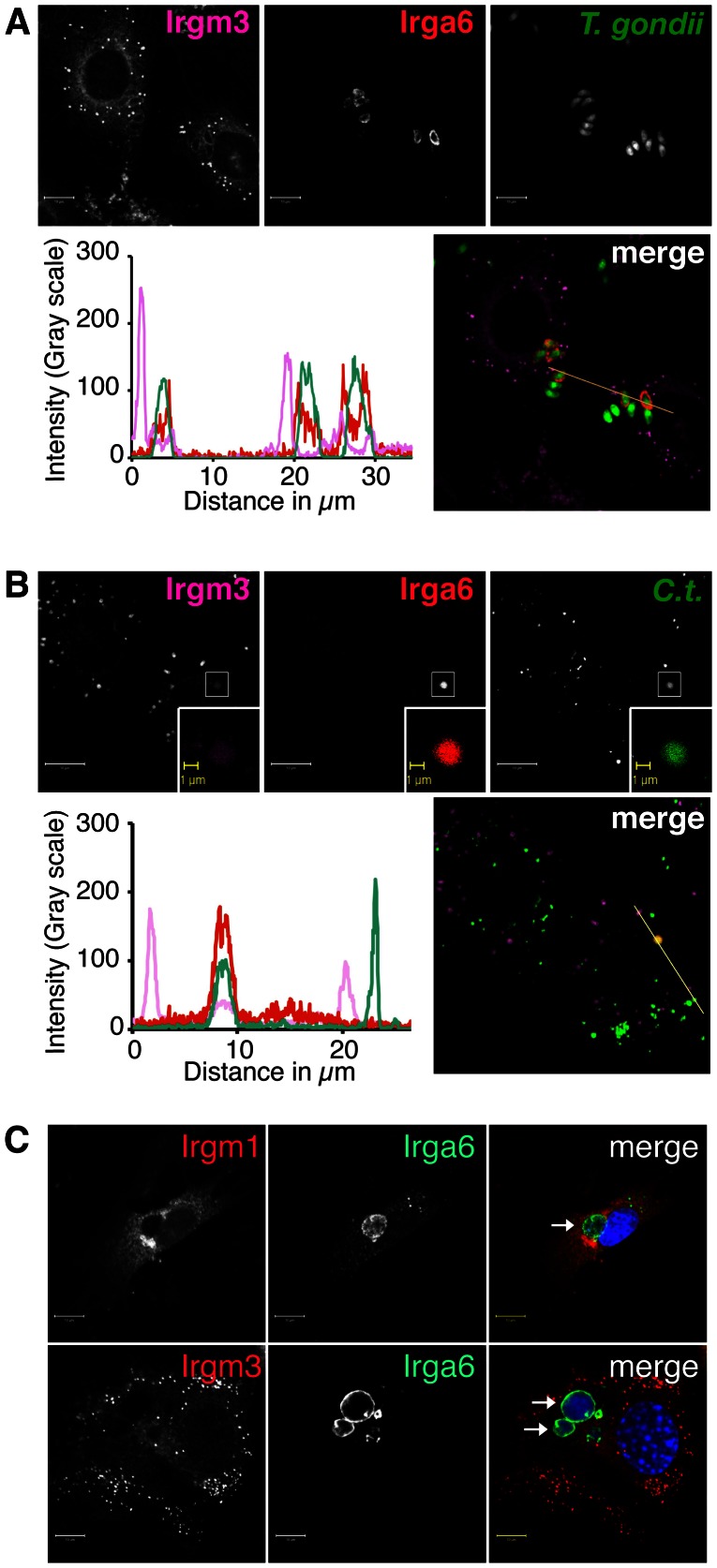
*T.* *gondii* PVs and *C. trachomatis* inclusions are largely devoid of Irgm1/m3. Images and line tracing of fluorescent intensity are shown. Data are representative of results obtained in at least 3 independent experiments conducted for each experimental design. (**A**). IFNγ-activated MEFs were infected with GFP-expressing *T. gondii* and stained with an anti-Irga6 and anti-Irgm3 antibody. (**B**) IFNγ-activated MEFs were infected with GFP-expressing *C. trachomatis* and stained with an anti-Irga6 and anti-Irgm3 antibody at 2 hpi. (**C**) MEFs were infected with *C. trachomatis* and activated with 100 U/ml of IFNγ at 3 hpi. Cells were fixed at 20 hpi and stained with an anti-Irga6 antibody in combinations with either anti-Irgm1 or anti-Irgm3 antibodies and Hoechst. Whereas Irga6 co-localized with inclusions at the expected frequency of 10–15% (data not shown), no targeting of endogenous Irgm1 or Irgm3 to inclusions was observed in the course of examining the staining of more than one hundred randomly selected inclusions. Arrows point at inclusions.

**Figure 5:**
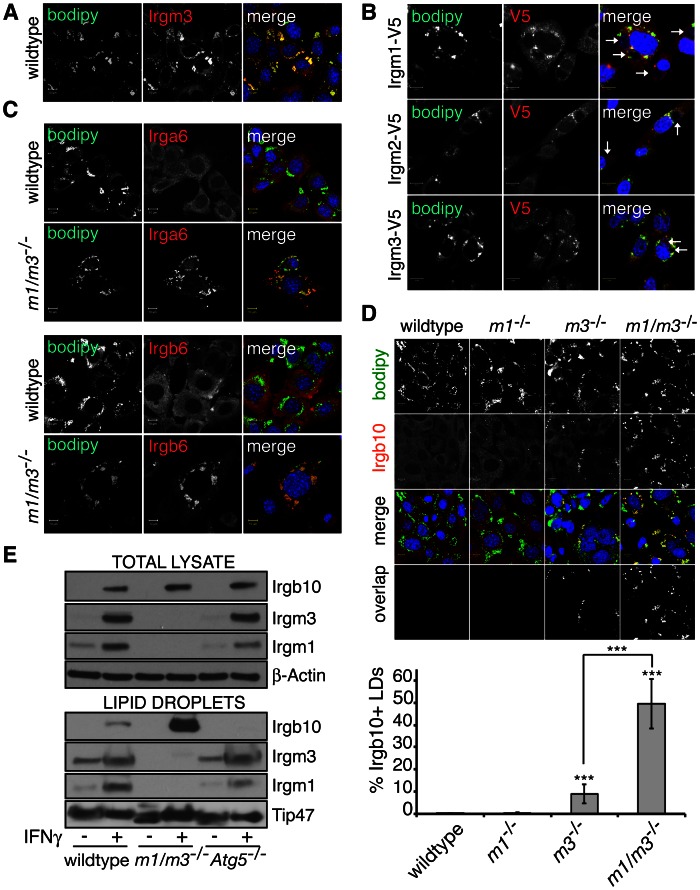
GKS proteins are enriched on LDs in IRGM-deficient cells. (**A**) Wildtype MEFs were treated overnight with OA and IFNγ and immunostained for endogenous Irgm3 and for LDs using BODIPY. (**B**) Wildtype MEFs were transfected with the three mouse paralogs Irgm1-3 tagged with V5, infected with *C. trachomatis* and treated with OA and IFNγ at 3 hpi. Cells were fixed at 20 hpi and stained with anti-V5, BODIPY and Hoechst. White arrows point at inclusions. (**C**) The localization of the GKS proteins Irga6 and Irgb6 were visualized in wildtype and *Irgm1/m3^−/−^* MEFs treated overnight with OA and IFNγ. (**D**) Cells were treated overnight with OA and IFNγ and stained with anti-Irgb10, BODIPY and Hoechst. Representative images are shown. Overlap in fluorescence signals derived from anti-Irgb10 and BODIPY stains is shown for these representative images. Data were analyzed as described in Materials and Methods and representative data of three independent experiments are shown. Statistical significance of group values relative to wildtype and between marked groups is shown (***, p<0.005). (**E**) LDs and total cell lysate were obtained from wildtype, *Irgm1/m3^−/−^* and *Atg5* ^−/−^ MEFs treated overnight with OA +/− IFNγ and analyzed by immunoblotting.

It is currently unknown where inside a cell IRGM proteins interact with GKS proteins to regulate their function. To determine whether IRGM proteins regulate GKS proteins directly at PV membranes, we first monitored the subcellular localization of IRGM proteins in cells infected with either *T. gondii* or *C. trachomatis*. As reported previously [14], [23], we found that endogenous Irgm3 but not Irgm1 associated with *T. gondii* PVs, albeit only weakly relative to its association with endogenous, puncta-like structures (Figure 4A and data not shown). These results are also in agreement with a previous report demonstrating that Irgm3 associates with *T. gondii* PVs at a lower frequency than GKS proteins do [24]. Next we examined the subcellular localization of endogenous Irgm1 and Irgm3 in *C. trachomatis*-infected cells. In agreement with a previous report [25], we detected association of Irgm3 with *C. trachomatis* inclusions at 2 hpi. However, Irgm3 associated only weakly with inclusions relative to its interactions with endogenous structures (Figure 4B). Similar to the staining pattern of *T. gondii* PVs, we failed to detect the presence of Irgm1 on inclusions (data not shown). To determine whether Irgm1 or Irgm3 could target established inclusions, we infected MEFs with *C. trachomatis* and subsequently treated cells with IFNγ at 3hpi. Under these experimental conditions endogenous as well as ectopically expressed Irgm1 and Irgm3 were not present at inclusion membranes in detectable amounts at 20 hpi (Figure 4C, Figure 5B and data not shown). Collectively, these data show that PVs formed by either *C. trachomatis* or *T. gondii* are devoid of substantial amounts of Irgm1 and Irgm3 proteins.

Because established PVs lack sizeable amounts of Irgm1/m3, we considered the hypothesis that IRGM proteins regulate Irgb10 and other GKS proteins at sites distinct from PVs. It is known that IRGM proteins localize to various endomembranes, including LDs, a neutral lipid storage organelle [6]. Specifically, Irgm3 was shown to localize to LDs in IFNγ-treated dendritic cells [26]. To determine whether or not Irgm3 also localizes to LDs in IFNγ-treated MEFs, we induced the formation of LDs by supplementing the growth media with oleic acid (OA) and subsequently stained these cells with the neutral lipid dye BODIPY493/503 and with anti-Irgm3 antibody. We found that Irgm3 co-localized with the BODIPY dye in IFNγ-treated MEFs (Figure 5A). To determine whether additional IRGM proteins localize to LDs, we monitored the localization of C-terminally V5-tagged Irgm1, Irgm2 and Irgm3 inside OA-treated MEFs. In addition to Irgm3-V5, Irgm1-V5 and Irgm2-V5 co-localized with a subset of LDs but not with inclusions (Figure 5B and Figure S1). Staining for endogenous protein confirmed the presence of Irgm1 but not Irgm2 on a subset of LDs (Figure S2 and data not shown). We next asked if GKS proteins were also found on LDs by immunostaining IFNγ-activated MEFs with antibodies directed against three representative GKS proteins Irga6, Irgb6 and Irgb10. We were unable to detect co-localization of these proteins with BODIPY-labeled LDs in wildtype MEFs by immunofluorescence (Figure 5C and D).

We independently confirmed these observations by assessing the levels of IRG proteins on purified LDs. LDs purified from IFNγ-treated, wildtype MEFs by sucrose gradient centrifugation displayed significant levels of Irgm1 and Irgm3 (Figure 5E), and relatively small amounts of Irgb10 (Figure 5E), suggesting possible transient interactions between IRGM proteins and Irgb10 on the surface of LDs. In summary, these data indicate that LDs of wildtype cells are decorated with Irgm1 and Irgm3 but only weakly associate with GKS proteins.

### GKS proteins mislocalize to LDs in *Irgm1/m3^−/−^* cells

Although LDs could play an essential role in guiding GKS proteins to inclusions, we thought this was unlikely, because LD-deficient cells lines still target Irgb10 to inclusions (H.A.S. and R.H.V., unpublished data). Because IRGM proteins inhibit GTP acquisition by GKS proteins and are believed to thereby block the ability of GKS proteins to bind lipids [15], [16], we formed an alternative hypothesis in which LD-resident IRGM proteins would prevent GKS proteins from binding to LDs. To test our hypothesis, we examined the localization of Irgb10 in *Irgm1/m3^−/−^* MEFs that contain IRGM-deficient LDs. We found that the LDs of *Irgm1/m3^−/−^* MEFs were heavily decorated with Irgb10 (Figure 5D). Targeting of Irgb10 to LD in *Irgm1/m3^−/−^* MEFs was primarily due to the absence of Irgm3, because *Irgm3* ^−/−^ MEFs but not *Irgm1* ^−/−^ MEFs displayed a substantial increase in the number of Irgb10-positive LDs (Figure 5D). The simultaneous deletion of both *Irgm3* and *Irgm1*, however, exacerbated the association of Irgb10 with LDs (Figure 5D) suggesting that these proteins fulfill partially redundant functions in protecting LDs against Irgb10 targeting. The role of Irgm1 and Irgm3 in guarding LDs was not limited to Irgb10 but extended to other GKS proteins including Irga6 and Irgb6 (Figure 5C). Again, Irgm3 was predominantly responsible for guarding LDs, because ectopic expression of Irgm3 in either *Irgm3* ^−/−^ or *Irgm1/m3^−/−^* MEFs prevented deposition of Irga6 on LDs (Figure S3). Irgm1 and Irgm3 were also required to prevent Irgb10 accumulation on LD in primary macrophages, indicating that the observed phenomenon is not cell type specific (Figure S4). Furthermore, endogenous LDs found infrequently in MEFs not treated with OA also acquired Irgb10 in the absence of Irgm1 and Irgm3 (Figure S5A), demonstrating that the aberrant localization of Irgb10 was not induced by OA treatment. Lastly, consistent with our immunofluorescence observations, we detected a robust increase in the amount of Irgb10 protein present in the LD fraction derived from *Irgm1/m3^−/−^* MEFs compared to wildtype MEFs (Figure 5E). These data combined demonstrate that GKS proteins target LDs in the absence of Irgm1/m3.

Because IRGM proteins can act as positive regulators of autophagy [8], [10], [27], we also considered the possibility that the mislocalization of GKS proteins to LDs in *Irgm1/m3^−/−^* cells was a consequence of disrupted autophagy. To test this hypothesis, we examined the subcellular localization of Irgb10 in autophagy-deficient *Atg5* ^−/−^ MEFs. We did not observe an increase in the association of Irgb10 protein with LDs in *Atg5* ^−/−^ MEFs (Figure 5E and Figure S6), indicating that a defect in autophagy is not the underlying cause for the mislocalization of GKS proteins to LDs in *Irgm1/m3^−/−^* MEFs.

Next, we asked whether IRGM proteins exclusively guard LDs. We observed that Irgb10 formed “aggregate-like structures” in *Irgm1/m3^−/−^* cells that did not identify as LDs (Figure S5A), suggesting that GKS protein could target additional “self” structures in the absence of Irgm1/m3. In support of this hypothesis, we found that GKS proteins also targeted mitochondria (Figure S5B) and peroxisomes (Figure S5C) in *Irgm1/m3^−/−^* cells. In wildtype cells mitochondria are decorated with Irgm1 (G.A.T., manuscript in preparation) and subsets of peroxisomes stain positive for Irgm3 (Figure S5D). In summary, our data suggest that IRGM proteins guard LDs and other organelles against the stable association with GKS proteins.

### A GTP-locked Irgb10 mutants targets IRGM-positive LDs

**Figure 6:**
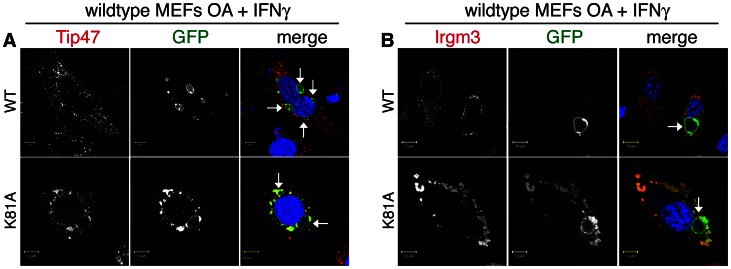
A GTP-locked Irgb10 mutant targets Irgm3-positive LDs in wildtype MEFs. Wildtype MEFs were transfected with GFP-tagged versions of Irgb10^WT^ and Irgb10^K81A^. Cells were treated overnight with OA, infected with *C. trachomatis* and treated with IFNγ at 3 hpi. At 20 hpi cells were stained for (**A**) the LD marker Tip47 and DNA or (**B**) Irgm3 and DNA. Representative images are shown. Arrows point at Hoechst-stained inclusions.

We demonstrated that endogenous GKS proteins like Irgb10 stably associate with LDs in the absence of IRGM proteins (Figure 5). Similarly, ectopically expressed Irgb10 frequently targets LDs in *Irgm1/m3^−/−^* MEFs (Figure S7A) but not in wildtype MEFs (Figure 6A). Two distinct models could explain the differential targeting of Irgb10 and other GKS proteins to IRGM-deficient but not IRGM-positive LDs: in the first model, the presence of IRGM proteins alters the molecular properties of LDs such that LDs do not serve as binding substrates for GKS proteins; in the second model, IRGM proteins directly interact with GKS proteins on the surface of LDs and block lipid binding. Previous studies have shown that IRGM proteins can transiently interact with GKS proteins and thereby retain GKS proteins in the GDP-bound, inactive state [15], [16], thus supporting the second model. We therefore predicted that an Irgb10 variant locked in the active, GTP-bound state should be able to overcome IRGM protein mediated restrictions on lipid binding and be able to target IRGM-positive LDs. To generate a GTP-locked Irgb10 mutant, we replaced the lysine residue of the conserved GKS motif with alanine (Irgb10^K81A^), as homologous mutations in Irga6 or Irgb6 interfere with GTP hydrolysis and force these GTPases into a GTP-locked state [16]. Similar to previous observations demonstrating the targeting of GTP-locked Irga6 to *T. gondii* vacuoles [16], we found that Irgb10^K81A^ co-localized with *C. trachomatis* inclusions (Figure 6). However, in contrast to Irgb10^WT^, Irgb10^K81A^ co-localized with Tip47-positive LDs (Figure 6A) and Irgm3 (Figure 6B) in wildtype MEFs. In contrast to Irgb10^WT^ and Irgb10^K81A^, a mutant with low affinity binding for GTP (Irgb10^S82N^) failed to associate with either inclusions or LDs (Figure S7). Overall, these data are consistent with a model, in which IRGM proteins on LDs block the activation of endogenous Irgb10 on the surface of LDs and prevent their stable association of Irgb10 with LDs.

### GBP proteins target IRGM-deficient LDs

**Figure 7:**
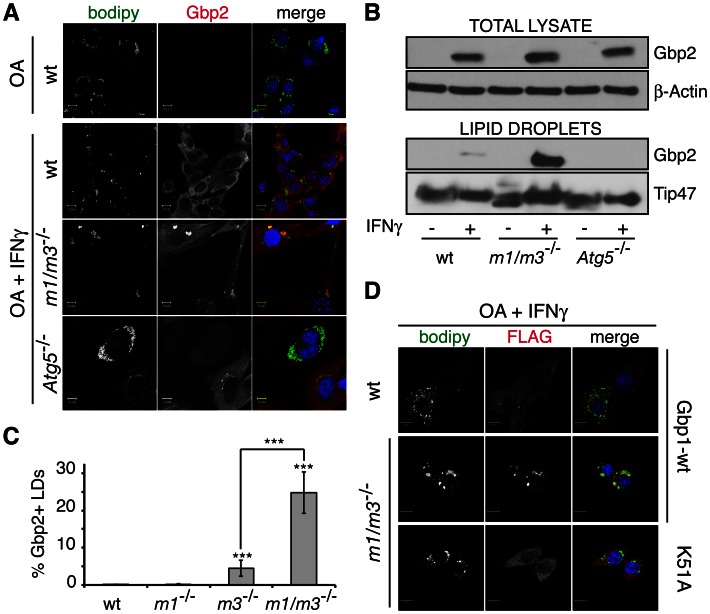
GBP proteins are enriched on IRGM-deficient LDs. MEFs of the indicated genotypes were treated overnight with OA alone or with OA and IFNγ. (**A**) MEFs were stained for endogenous Gbp2 and LDs using BODIPY (**B**) LDs and total cell lysate were obtained from wildtype, *Irgm1/m3^−/−^* and *Atg5* ^−/−^ MEFs and analyzed by immunoblotting. Here, the same lysates and LD preparations were used as in Figure 5E. The data are representative of three independent LD preparation experiments. (**C**) Quantitative analyses of Gbp2 co-localization with LDs were done using MBF-ImageJ software as described in Materials and Methods. Data are representative of three independent experiments. Statistical significance of group values relative to wildtype and between marked groups is shown (***, p<0.005). Corresponding representative images are shown in Figure S8. (**D**) FLAG-tagged Gbp1^WT^ and Gbp1^K51A^ mutant were expressed in MEFs treated overnight with OA and IFNγ. BODIPY and anti-FLAG staining was used to monitor localization of Gbp1 to LDs.

GBP proteins constitute a second large family of IFNγ-inducible GTPases known to target PVs and to provide resistance to infections with vacuolar pathogens [3]. Because we previously observed that the subcellular location of the GBP protein Gbp2 is altered in the absence of IRGM proteins [27], we hypothesized that IRGM proteins could guard self-membranes against the improper deposition of not only GKS but also GBP proteins. Consistent with this, we found that Gbp2 co-localized with LDs in *Irgm1/m3^−/−^* but not in wildtype MEFs (Figure 7A) and was enriched in LD fractions obtained from *Irgm1/m3^−/−^* cells (Figure 7B). Irgm1 and Irgm3 appeared to fulfill partially redundant functions in guarding LDs against Gbp2 targeting, because Gbp2 localization to LDs was more pronounced in *Irgm1/m3^−/−^* MEFs than in *Irgm1* ^−/−^ or *Irgm3* ^−/−^ single gene deletion cells (Figure 7C and Figure S8). Because both Irgm1 and Irgm3 regulate the formation and/or maturation of autophagosomes [8], [27], it was formally possible that the mislocalization of Gbp2 to LDs in *Irgm1/m3^−/−^* cells resulted from a defect of these cells in autophagy. However, it is unlikely that defective autophagy is the primary cause for mislocalization of Gbp2 to LDs, because LDs inside autophagy-deficient *Atg5* ^−/−^ MEFs remained exempt from Gbp2 targeting (Figure 7A and B).

Similar to endogenous Gbp2, we found that ectopically expressed, N-terminally tagged FLAG-Gbp1 protein was redirected to LDs in the absence of Irgm1 and Irgm3 proteins (Figure 7D). To determine whether activation of Gbp1 was critical for targeting IRGM-deficient LDs, we expressed a FLAG-Gbp1^K51A^ mutant form that has previously been shown to be defective for nucleotide binding and protein oligomerization [28]. In contrast to wildtype FLAG-Gbp1, we found that FLAG-Gbp1^K51A^ failed to associate with LDs in *Irgm1/m3^−/−^* cells (Figure 7D). These data suggest that the active form of Gbp1 associates with IRGM-deficient LDs.

### The known Gbp1 effector p62 targets IRGM-deficient LDs for degradation

**Figure 8:**
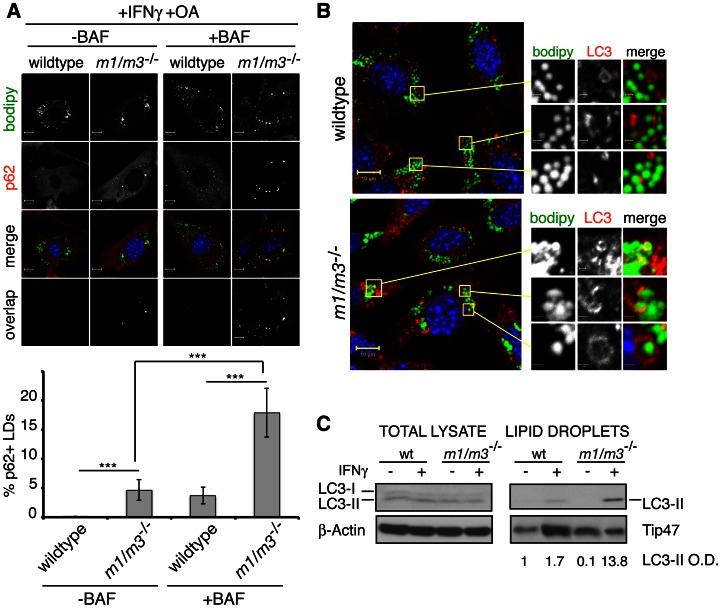
IRGM-deficient LDs recruit p62 and LC3-II. (**A and B**) Wildtype and Irgm1/m3^−/−^ MEFs were treated overnight with OA and IFNγ and stained with BODIPY and anti-p62 or anti-LC3, respectively. Representative images of at least 3 independent experiments are shown. Where indicated, cells were treated with Bafilomycin (BAF). (**A, lower panel**) Quantitative analyses of p62 co-localization with LDs were done using MBF-ImageJ software as described in Materials and Methods (***, p<0.005). (**C**) Total cell lysates and LD protein preparations from the indicated samples were analyzed for LC3 protein expression. Densitometric analyses for protein quantification were carried out using ImageJ 1.45 s software. The relative optical density (O.D.) for LC3-II expression normalized against expression of the LD marker Tip47 is listed below the corresponding lanes in arbitrary units.

Our data demonstrated that Gbp1 and Gbp2 localized to IRGM-deficient LDs. One of the known effector molecules of Gbp1 is the autophagic adaptor protein p62/sequestosome-1 [29]. We therefore hypothesized that Gbp1 proteins residing on IRGM-deficient LDs would be able to recruit p62 to LDs. In support of our hypothesis we found that 4–5% of LDs in IFNγ-treated *Irgm1/m3^−/−^* cells stained positive for p62 (Figure 8A). In contrast to *Irgm1/m3^−/−^* cells, we were unable to detect p62 on LDs of IFNγ-treated wildtype cells using immunofluorescence microscopy (Figure 8A).

A critical function of p62 is to bind to macromolecular cargo that is destined for autophagic destruction [30]. To deliver its cargo to autophagosomes, p62 also binds directly to the ubiquitin-like protein LC3, a maker of autophagosomes. To determine whether IRGM-deficient LDs are delivered to autophagosomes upon IFNγ activation, we incubated both wildtype and *Irgm1/m3^−/−^* MEFs with OA and IFNγ and subsequently stained cells with anti-LC3 and BODIPY. In these experiments, we frequently observed LDs that were engulfed within ring-like LC3-positive structures in *Irgm1/m3^−/−^* but not in wildtype MEFs (Figure 8B). Similarly, LDs purified from *Irgm1/m3^−/−^* cells were enriched for LC3-II, the lipidated form of LC3 that is associated with autophagosomes (Figure 8C). Collectively, these data strongly suggested that IRGM-deficient LDs were captured inside autophagosomes upon IFNγ activation.

To test this model further, we treated cells with the lysosomotropic H^+^-ATPase inhibitor bafilomcyicn (BAF), a known inhibitor of autophagic flux [31]. We observed a substantial increase in the number of p62-positive LDs in *Irgm1/m3^−/−^* MEFs upon combined treatment with IFNγ and BAF (Figure 8A). BAF treatment also resulted in the appearance of p62-positive LDs in IFNγ-treated wildtype MEFs, however, at a frequency significantly lower than what we observed in BAF-treated *Irgm1/m3^−/−^* MEFs (Figure 8A). These data indicated that the targeting of p62 to IRGM-deficient LDs resulted in the degradation of LD-bound p62. Furthermore, our observations excluded an alternative model in which the increase in the number of p62-positve LDs in *Irgm1/m3^−/−^* MEFs was due to a defect in autophagosome maturation in these cells.

**Figure 9:**
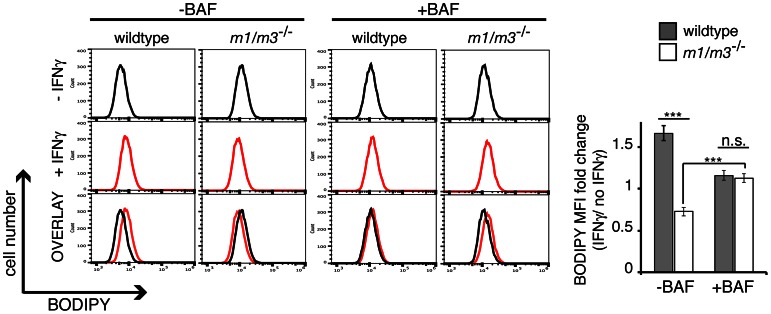
IFNγ induces degradation of LDs in Irgm1/m3^−/−^ MEFs. Cells were cultured overnight in the absence or presence of IFNγ and in the absence of OA. 12 hours post IFNγ treatment, BAF was added to the culture media as indicated for an additional 8–12 hours and the BODIPY signal was measured by flow cyometry. Flow cytometry histograms of BODIPY-labeled wildtype and Irgm1/m3^−/−^ MEFs representative of three independent experiments are shown. The fold change in the average mean fluorescent intensity (MFI) in response to IFNγ treatment is plotted in the panel on the right (***, p<0.005).

We then asked whether the increased association of p62 and LC3 with IRGM-deficient LDs would affect the total mass of LDs. To quantify LD mass, we used a flow cytometry approach using BODIPY staining, as previously described [26]. We found that IFNγ treatment resulted in an increase in the BODIPY signal in wildtype MEFs, similar to the observations previously made in dendritic cells [26]. In contrast to the increase in the BODIPY signal observed in IFNγ-treated wildtype cells, the BODIPY signal decreased in IFNγ-treated *Irgm1/m3^−/−^* MEFs (Figure 9 and Figure S9), suggesting increased rates of LD degradation in IRGM-deficient cells. To determine whether the decrease in LD mass in *Irgm1/m3^−/−^* MEFs was due to autophagy ( = lipophagy), we treated cells with BAF. BAF treatment blocked the IFNγ-induced decrease in LD mass in *Irgm1/m3^−/−^* MEFs (Figure 9). In sum, these data strongly support a model in which p62 targets IRGM-deficient but not IRGM-guarded LDs and delivers IRGM-deficient LDs to autophagosomes for degradation.

### IRGM proteins facilitate GBP protein targeting to *C. trachomatis* inclusions and *T. gondii* PVs

**Figure 10:**
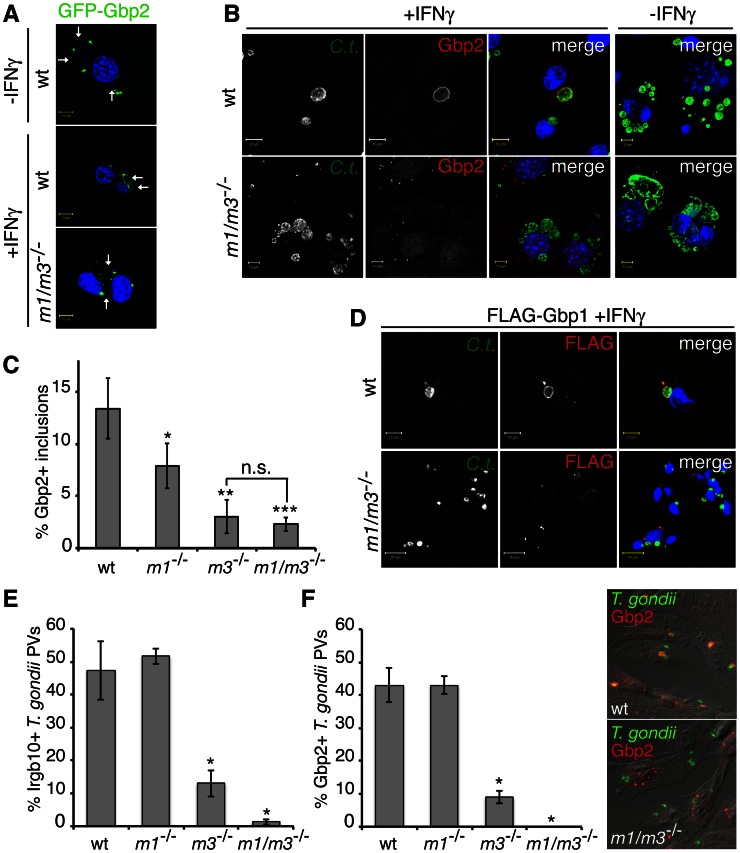
Irgm1 and Irgm3 are critical co-factors for the targeting of GBP proteins to *C.* *trachomatis* inclusions and *T. gondii* PVs. (**A**) Localization of ectopically expressed GFP-Gbp2 was monitored in *C. trachomatis* infected MEFs at 20 hpi. Arrows point at Hoechst-stained inclusions (**B**). Wildtype and *Irgm1/m3^−/−^* MEFs were infected with *C. trachomatis*, left untreated or treated with IFNγ at 3 hpi. Cells were fixed at 20 hpi and stained with anti-*C. trachomatis*, anti-Gbp2 and Hoechst. (**C**) Co-localization of Gbp2 with inclusions in wildtype and *Irgm1/m3^−/−^* MEFs was quantified as described in Materials and Methods. Error bars represent standard deviations of three independent experiments. Statistical significance of group values relative to wildtype and between marked groups is shown (*, p<0.05; **, p<0.01; and ***, p<0.005). (**D**) MEFs were transfected with FLAG-Gbp1, infected with *C. trachomatis* and activated with IFNγ at 3 hpi. Cells were fixed at 20 hpi and stained with anti-FLAG and anti-*C. trachomatis*. Representative images are shown. (**E**) MEFs were treated overnight with 200 U/ml of IFNγ prior to infections. Localization of endogenous Irgb10 to *T. gondii* PVs was monitored at 0.5 hpi. Data are representative of three independent experiments (*, p<0.05 relative to wildtype). (**F**) Localization of endogenous Gbp2 to *T. gondii* was monitored at 0.5 hpi. Data are representative of three independent experiments (*, p<0.05 relative to wildtype). Representative epifluorescent images of *T. gondii*-infected wildtype and *Irgm1/m3^−/−^* MEFs are shown.

It has previously been reported that targeting of GBP proteins to *T. gondii* PVs is facilitated by unknown IFNγ-inducible factors [32], [33]. Because our data had already established functional interactions between GBP and IRGM proteins, we asked whether IRGM proteins could act as IFNγ-inducible co-factors promoting the recruitment of Gbp2 to PVs. We found that ectopically expressed Gbp2-GFP fusion proteins failed to localize to *C. trachomatis* inclusions in the absence of IFNγ treatment or in IFNγ-activated *Irgm1/m3^−/−^* cells (Figure 10A). Similarly, we observed that both Irgm1 and Irgm3 played critical roles in facilitating targeting of endogenous Gbp2 protein to inclusions (Figure 10B and C). Similar to Gbp2, recruitment of FLAG-Gbp1 to inclusions was also dependent on IRGM proteins (Figure 10D). To determine whether the regulatory role of IRGM proteins extends to the recruitment of GBP proteins to PVs formed by pathogens other than *C. trachomatis*, we monitored co-localization of both Gbp2 and, as a control, Irgb10 with *T. gondii* vacuoles in wildtype, *Irgm1* ^−/−^, *Irgm3* ^−/−^ and *Irgm1/m3^−/−^* MEFs. We observed that the deletion of both *Irgm1* and *Irgm3* caused a near complete defect in the recruitment of Irgb10 (Figure 10E) and Gbp2 to *T. gondii* PVs at 0.5 hpi (Figure 10F). These observations demonstrate that the expression of IRGM proteins is critical for the efficient delivery of GBP proteins to vacuoles formed by distinct intracellular pathogens.

## Discussion

**Figure 11:**
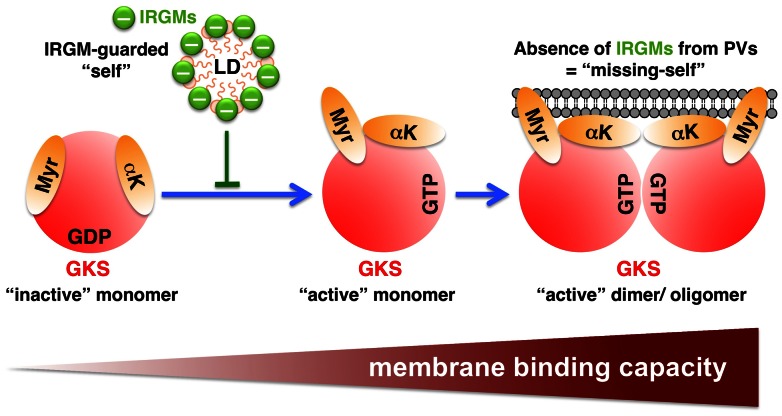
Proposed model: IRGM proteins regulate the localization of GKS and GBP proteins away from “self” membranes and towards “non-self” PVs. Upon GTP acquisition ( = “activation”), GKS and GBP proteins transition into a conformational state that permits lipid binding. Protein oligomerization of activated GKS and GBP proteins further increases their membrane-binding activity and is critical for the stable association of GKS and GBP proteins with most membranes. IRGM proteins negatively regulate the “activation” and subsequent oligomerization of GKS and GBP proteins. Accordingly, IRGM-positive membranes/micelles (e.g. LDs) are protected against GKS and GBP binding and IRGM-free membranes (e.g. surrounding PVs) are targets for GKS and GBP binding. In addition to protecting self-structures, IRGM proteins also maintain a pool of inactive, monomeric GKS and GBP proteins. Monomeric GKS and GBP proteins are cytosolic and move around inside the cell until they encounter PVs. In Irgm1/m3^−/−^ cells, the binding to IRGM-deficient endomembranes decreases the pool of available, monomeric GKS and GBP proteins and therefore diminishes the targeting of GKS and GBP proteins to PVs. In this illustration, the GKS protein Irgb10 with its two the putative lipid binding domains is given as an example (Myr = myristoyl group; αK = amphipathic helix).

The data presented in this study support a model in which Irgm1 and Irgm3 proteins act as “guard molecules” that block GKS and GBP proteins from stably associating with “self” structures (Figure 11). On PVs, however, guarding Irgm1 and Irgm3 proteins are present at such low levels that GKS and GBP proteins can firmly attach to these unprotected membranes. In support of our model we found that Irgm1 and Irgm3, but not GKS and GBP proteins, are present in LDs of wild type cells (Figure 5 and Figure 7). In the absence of Irgm1 and Irgm3, however, normally GKS-/GBP-deficient LDs become decorated with various GKS and GBP proteins. We provide evidence that GKS and GBP proteins assemble on IRGM-deficient LDs in their GTP-bound, i.e. “active” state (Figure 7 and Figure S7). According to our model GKS-/GBP-decorated LDs should resemble GKS-/GBP-decorated PVs and would therefore be expected to become targets of GKS-/GBP-solicited immune responses. Consistent with such a scenario, we demonstrate that the Gbp1 effector protein p62 is recruited to IRGM-deficient LDs. The targeting of p62 to LDs in *Irgm1/m3^−/−^* MEFs likely accounts for the enhanced association of IRGM-deficient LDs with the autophagic marker LC3-II and the decrease in LD mass upon IFNγ activation that we observed in *Irgm1/m3^−/−^* MEFs. Our observations are consistent with a previous report that showed that the number of LDs is significantly reduced in IFNγ-activated *Irgm3^−/−^* dendritic cells compared to IFNγ-activated wildtype dendritic cells [26]. Whereas the authors of this previous study speculated that Irgm3 could play a role in the neoformation of LDs triggered upon IFNγ receptor signaling, our data strongly suggest that the decrease in LDs observed in IRGM-deficient cells primarily results from GBP-mediated autophagy of LDs. However, because our experiments were conducted in MEFs, additional studies are needed to determine whether Irgm3 may also play a role in LD neoformation in dendritic or other cell types.

While we propose that IRGM proteins guard self-organelles against misdirected attacks by GKS and GBP proteins, our studies do not exclude additional roles for IRGM proteins in organelle homeostasis. For example, human IRGM protein translocates to mitochondria and induces mitochondrial fission [10]. Because mitochondrial fission not only results in the production of radical oxygen species and the induction of antimicrobial autophagy, but also contributes to the isolation and removal of damaged segments of mitochondria, IRGM proteins may indeed regulate the homeostasis of specific organelles like mitochondria.

The question now arises as to why the “guarding” Irgm1 and Irgm3 proteins are present on “self” membranes but largely absent from “non-self” PVs. The answer to this question may be quite obvious, if one considers that IRGM proteins can exert antimicrobial activities directly, once localized to PVs [3]. To escape from IRGM-mediated antimicrobial activities like lysosomal targeting, we propose that vacuolar pathogens have evolved strategies to actively avoid co-localization with IRGM proteins. The absence of Irgm1/m3 from PVs would initially allow pathogens to establish a vacuolar niche permissive for microbial replication. However, the evasion of IRGM proteins would simultaneously mark PVs for immune targeting by GKS and GBP proteins. The principle underlying this type of intracellular immune recognition is similar to the extracellular immune recognition process by which NK cells detect transformed and/or virus-infected cells [34]. NK cells express inhibitory receptors on their cell surface. The ligands for one set of inhibitory NK receptors are MHC class I molecules displayed on the surface of host cells. The primary function of the MHC class I molecules is to display viral or tumor antigens to cytotoxic T cells. To avoid immune recognition by cytotoxic T cells, many tumor cells and viruses have evolved mechanisms to downmodulate MHC class I surface expression. However, the failure of MHC class I-deficient cells to provide an inhibitory signal to NK cells, allows NK cells to recognize the *missing of “self”* MHC class I in transformed or infected cells. In this analogy IRGM proteins resemble MHC class I molecules: just like MHC class I molecules, IRGM proteins fulfill dual functions in that they can promote antimicrobial activities directly and simultaneously act as inhibitory molecules that block the activation of an alternative defense system.

How do Irgm1 and Irgm3 proteins guard membranes against GKS and GBP proteins? Studies performed by Howard and colleagues indicate that IRGM proteins act as Guanine nucleotide Dissociation Inhibitors (GDI) for GKS proteins [15], [16]. Based on these findings, Howard and colleagues proposed that by maintaining GKS GTPases in the GDP-bound, monomeric state, IRGM proteins reduce the lipid binding capacity of GKS proteins and block their stable association with IRGM-coated membranes. Here, we provide direct evidence in support of this model. As originally proposed by Hunn et al., our data indicate that the absence of IRGM proteins from PVs promotes the transition of GKS proteins into the GTP-bound, active state and their stable association with IRGM-deficient PVs. We show here that IRGM-deficient membranes are also targets for GBP proteins (Figure 7). Whether IRGM proteins act as GDIs for GBP proteins or block the ability of GBP proteins to associate with LDs by an alternative mechanism will need to be elucidated in future studies.

The lipid binding substrates for GKS and GBP proteins are currently unknown. However, lipid components that are present in LDs as well as in *T. gondii* parasitophorous and *Chlamydia* inclusion membranes are obvious candidates to act as GKS- and GBP-interacting molecules. It is tempting to speculate that GKS and GBP proteins might have evolved to preferentially bind to lipids that are frequently found in PVs but infrequently found on the cytosolic face of most endomembranes [35]. According to this model, most “self” structures would be protected against the erroneous attack by GKS and GBP proteins for two reasons: 1) the presence of guarding IRGM proteins and 2) the relative sparsity of lipid binding substrates on the cytosolic leaflet of “self”-membranes. This model would suggest that GKS and GBP proteins tether specifically to PVs due to the missing of “self” IRGM proteins from PVs and the presence of an unknown “second signal” on PVs. As suggested above, a unique pattern of lipids may provide such a second signal, although other molecules may also be involved. Albeit speculative at this point, we propose that LDs feature such a second signal and therefore become primary targets for GKS and GBP proteins in the absence of IRGM guard molecules.

The requirement of a second signal for GKS/GBP membrane targeting as proposed in the model outlined above could in part explain why *Irgm1/m3^−/−^* mice and cells are viable in spite of lacking two critical “guard” proteins. Alternatively, expression of Irgm2 in *Irgm1/m3^−/−^* cells may provide sufficient protection to assure survival of *Irgm1/m3^−/−^* cells upon immune activation. This second model would necessitate that Irgm2 like Irgm1/m3 guards “self” membranes against GKS and GBP proteins.

In addition to guarding self-structures, expression of IRGM proteins is required for the efficient targeting of endogenous GKS proteins to *C. trachomatis* inclusions (Figure 3) and *T. gondii* PVs [16]. However, tetramerized Irgb10-dsRED, when overexpressed, targets PVs efficiently in IRGM-deficient cells (Figure 2A). These data argue against a direct role for IRGM proteins in delivering GKS proteins to PVs. We therefore propose a model in which IRGM proteins fulfill an indirect role in targeting endogenous GKS proteins to PVs: in this model GKS proteins can bind to an excess of unguarded “self” membranes in IRGM-deficient cells. Consequently, the cellular pool of available GKS proteins is diminished in IRGM-deficient cells and the efficiency of PV targeting is reduced.

In addition to GKS proteins, GBP proteins also bind to PVs. The delivery of GBP proteins to PVs requires the presence of a previously unknown IFN-inducible cofactor(s) [32], [33]. Here, we identify IRGM proteins as one such co-factor. We propose that IRGM proteins promote the recruitment of GBP proteins to PVs by a mechanism similar to that which regulates the subcellular localization of GKS proteins. GBP proteins bind to lipids as activated oligomers [36], [37] and GBP mutants deficient in GTP binding fail to localize to PVs [29], [33]. In this study, we demonstrate that IRGM proteins on LDs prevent GBP recruitment, suggesting that IRGM proteins interfere with the ability of GBP proteins to transition into the GTP-bound, oligomeric state. In support of this hypothesis, we found that Gbp2 forms high molecular weight aggregates in the absence of Irgm1/m3 (A.S.P. and J.C., unpublished results). Therefore, IRGM proteins may promote GBP recruitment to PVs by maintaining a pool of GDP-bound, monomeric GBP proteins that are able to diffuse to their target sites.

Additional evidence for functional interactions between the GBP and IRG protein families comes from the observation that one or more members of GBP protein family associate with Irgb6 in complexes [38]. Deletion of the chromosomal region containing the genes *Gbp1-3*, *Gbp5* and *Gbp7* causes a partial defect in the recruitment of Irgb6 and Irgb10 but not Irga6 to *T. gondii* PVs [38], suggesting that physical interactions between specific GBP proteins and Irgb6/b10 promote targeting of Irgb6/b10 to PVs. In contrast to the partial GKS targeting defects of *Gbp*-deficient cells, *Irgm1/m3^−/−^* cells display a nearly complete deficiency in recruiting either Irgb10 (Figure 10E) or Gbp2 protein to *T. gondii* PVs (Figure 10F). The combined results from both studies suggest that Irgm1 and Irgm3 regulate the recruitment of both GKS and GBP proteins to PVs, while one or more PV-targeted GBP proteins augment the recruitment of a subset of GKS proteins through direct physical interactions. In summary our data demonstrate that IRGM proteins orchestrate the proper targeting of antimicrobial GBP and GKS proteins away from “self” membranes and towards “non-self” PVs.

## Materials and Methods

### Host cell culture, bacterial and protozoan strains and infections

MEFs derived from wildtype, *Irgm1^−/−^*, *Irgm3^−/−^* and *Irgm1/m3^−/−^* mice were previously described [39], [40]. MEFs and African green monkey kidney Vero cells were cultured in Dulbecco's modified Eagle's medium supplemented with 10% heat-inactivated fetal bovine serum (FBS) (Denville and Life Technologies). Primary murine bone marrow macrophages were isolated from the tibia and femurs of 2- to 4-months-old mice as described before [41]. *C. trachomatis* LGV-L2 was propagated as described [39]. A previously described GFP-expression vector [42] was introduced into LGV-L2 for visualizing *C. trachomatis* at 2 hpi. GFP-expressing *Toxoplasma gondii* tachyzoites of the type II strain Prugniaud A7 were a generous gift from Dr. John Boothroyd (Stanford University, Stanford, CA) [43]. Infections with *C. trachomatis* were performed at a nominal multiplicity of infection of 1–5 as described [39]. For *T. gondii* infections cells were incubated overnight with or without 200 U/ml of IFNγ and asynchronously infected with tachyzoites at a nominal multiplicity of infection of 5–10 for thirty minutes.

### Induction of LD formation, LD purification and immunoblotting

For lipid loading experiments, OA (Sigma) was precomplexed with fatty acid-free BSA (Sigma) in PBS and emulsified by sonication. OA was added to growth media at final concentration of 100 µM for immunofluorescence experiments. LDs were isolated from MEFs as described before [44] with minor modifications as outlined here. Cells were grown in 150 mm dishes in DMEM +10% FBS and incubated with OA at 300 µM in the presence or absence of 100 U/mL of IFNγ for 14 h before harvesting LDs. Cells were washed with PBS and collected in 5 ml TNE buffer [20 mMTris-HCl (pH 7.5), 0.15 M NaCl, and 1 mM EDTA] containing protease inhibitors (Roche Diagnostics). Cells were lysed on ice with ∼30 strokes/150 mm dish in a Dounce homogenizer and 80 µl of total lysates were collected from each sample and stored at −20°C for Western blotting. Cell lysates were then adjusted to 0.45 M sucrose, overlaid with 2 ml each of 0.25 M, 0.15 M, and 0 M Sucrose/TNE and centrifuged at 30,000 rpm for 90 min at 4°C in an SW41 rotor (Beckman Coulter). The floating LD-enriched fat layer was collected, diluted in TNE, and refloated at 47,000 rpm for 45 min in a TLA55 rotor (Beckman Coulter). LDs were collected, and lipids were extracted with 4 volumes of diethyl ether. Delipidated proteins were precipitated with ice-cold acetone for 1 h, solubilized in 0.1%SDS and 0.1 N NaOH, and normalized for total protein content by Bradford assay before SDS-PAGE and immunoblot analysis. Following protein transfer to nitrocellulose membranes, membranes were incubated with antibodies as listed below. Densitometric analyses for protein quantification in Western blots were carried out using Image J 1.45 s software.

### Immunocytochemistry, BODIPY staining and data analysis

Immunocytochemistry was performed essentially as described previously [39], [44]. Cells were washed thrice with PBS, pH 7.4 prior to fixation. Cells were fixed with 3% formaldehyde and 0.025% glutaraldehyde for 20 min at room temperature (RT) in all experiments that visualized LDs. *T. gondii*-infected cells were fixed with 4% paraformaldehyde in PBS, pH 7.4, cells for 20 min at RT. In all experiments involving LD staining, fixed cells were permeabilized/blocked with 0.05% (v/v) saponin and 2% BSA/PBS (SBP) for 30 min at RT. When preserving LD structures was not required, fixed cells were permeabilized in 0.1% (v/v) Triton X-100 in PBS for ten minutes, blocked for 1 h with 2% (w/v) BSA (Equitech-Bio Inc.) in PBS, and then stained with various primary antibodies, followed by Alexa Fluor-conjugated secondary antibodies (Molecular Probes/Invitrogen). Working solutions of antibodies and BODIPY 493/503 (10 µg/ml) (Invitrogen) for immunofluorescence were prepared in SBP (for LD visualization) or in 2% (w/v) BSA/PBS (for all other experiments). Nucleic and bacterial DNA were stained with Hoechst 33258 according to the manufacturer's protocol. Mitochondria were visualized using MitoTracker Red CMXRos (Invitrogen) according to the manufacturer's instructions. Stained cells were washed with PBS, mounted on microscope slides with FluorSave (Calbiochem) or ProLong Gold (Invitrogen), and allowed to cure overnight. Cells were imaged using either a Zeiss LSM 510 inverted confocal microscope or a Zeiss Axioskop 2 upright epifluorescence microscope. Co-localization of proteins with PVs was quantified in at least 3 independent experiments. In each experiment at least ten randomly selected fields were imaged for each condition for each cell type. Differential interference contrast images were used to identify extracellular *T. gondii* tachyzoites because the vacuoles typically contained only one parasite under the experimental conditions used. The fraction of Gbp2- or Irgb10-positive vacuoles was determined for each field by dividing the number of Gbp2- or Irgb10-labeled vacuoles by the total number of vacuoles. Co-localization with *C. trachomatis* inclusions was quantified using the identical approach. Co-localization of Irgb10 and Gbp2 with LDs was quantified using MBF ImageJ software (developed by Wayne Rasband, National Institutes of Health, Bethesda, MD; available at http://rsb.info.nih.gov/ij/index.html). Images were pre-processed to correct uneven illumination and to minimize noise and background. The co-localization rates were measured based on Manders' coefficient, which varies from 0 to 1. A coefficient value of zero corresponds to non-overlapping images while a value of 1 reflects 100% co-localization between the images being analyzed. To perform line tracings, i.e. analyze the fluorescence signal intensity profiles of pixels along a selection from images, we used ImageJ software.

### LDs measurement by flow cytometry

Cells were treated with or without IFNγ (200 U/ml) in the absence or presence of OA for 20 to 24 hours. Where indicated, BAF was supplemented at a final concentration of 100 nM at 12 hours post IFNγ activation. LD mass was determined by Flow Cytometry as described elsewhere [26]. Briefly, after fixing the cells with 2% PFA, cells were stained with BODIPY 493/503 at 5 µg/ml in FACS buffer (PBS, 1% BSA and 0.1% NaN3) for 30 minutes and washed with FACS buffer prior to analysis.

### Antibodies

The primary antibodies used included anti-Irgm1 mouse monoclonal antibody 1B2 [11] at 1∶10; anti-Irga6 mouse monoclonal antibody 10D7 [12] at 1∶10; anti-Irgb10 rabbit polyclonal antiserum [39] at 1∶1000; anti-Irgb6 rabbit polyclonal antisera [27] at 1∶1000; anti-Irgm3 rabbit polyclonal antisera [45] at 1∶1000; mouse monoclonal anti-Irgm3 antibody (BD-Transduction Labs) at 1∶300; FITC-labeled mouse monoclonal anti-*C. trachomatis* MOMP [39] at 1∶200; rabbit anti-IncG [46] at 1∶50; anti-V5 mouse monoclonal antibody (Invitrogen) at 1∶1000; anti-FLAG mouse monoclonal antibody F1804 (Sigma) at 1∶500, rabbit anti-Pmp70 (abcam) at 1∶500; anti-TIP47 polyclonal antisera (Proteintech) at 1∶1000; anti-p62/SQSTM1 rabbit polyclonal antibody (MBL International) at 1∶500; and anti-LC3 rabbit polyclonal antibody (MBL International) at 1∶1000. An affinity-purified polyclonal rabbit anti-Gbp2 antibody was generated against the peptide EVNGKPVTSDEYLEHS of Gbp2 and used at 1∶1000.

### Cloning of expression constructs and cell transfection

An Irgb10-GFP expression construct has been previously described [39]. Site-directed mutagenesis was performed using the QuikChange II Site-Directed Mutagenesis Kit (Agilent Technologies Inc.) to introduce the listed point mutations and deletions into the same construct. Standard cloning techniques were used to generate to insert DNA encoding dsRED and the yeast protein TyA into the listed GFP expression constructs. DNA oligonucleotides used for cloning are listed in Table 1. A previously described TyA expression construct [21], a kind gift from Dr. Stephen Gould, was used as a template for DNA amplification. The C57BL/6J-derived cDNAs of Irgm1, Irgm2 and Irgm3 were cloned into pcDNA3.1/V5-His-TOPO (Invitrogen) following the manufacturer's instructions. FLAG-tagged and GFP-tagged expression constructs of Gbp1 and Gbp2 and Gbp1 mutant variants have been previously described [33]. MEFs were transduced using the MSCV-based delivery system (Clontech) or transfected using Attractene (Qiagen) following the manufacturers' instructions.

**Table 1:** DNA oligonucleotides.

b10K81AF	GGGAGACAGGGGCAGGGGCGTCCACGTTCATTAATGC
b10K81AR	GCATTAATGAACGTGGACGCCCCTGCCCCTGTCTCCC
b10S82NF	AGACAGGGGCAGGGAAGAACACGTTCATTAATGCCC
b10S82NR	GGGCATTAATGAACGTGTTCTTCCCTGCCCCTGTCT
b10ATGXhoIF	AATCTCGAGCCATGGGTCAGTCTTCTTC
b10CTDXhoIF	AATCTCGAGATGATGCCAGCACACAAGCGC
b10BamH1R	ATAGGATCCCCTCAGAGTCCACACTGTC
dsREDEcoR1F	TATGAATTCGTCGACATGGCCTCCTCCGAGGACG
dsREDEcoR1R	TATGAATTCCAGAAAACAGGTGGTGGCGG
dsREDHindIIIF	TATAAGCTTATGGCCTCCTCCGAGGACG
dsREDBamH1R	ATAGGATCCCAGAAAACAGGTGGTGGCGG
TyAMfeIF	AATCAATTGGAGAGCCAGCAGCTGAGCCAG
TyAEcoR1R	ATAGAATTCAGAGTTGTTGGAGGTGCTCAC
b10CTDBamH1F	AATGGATCCCCAGCACACAAGCGCCACATC
b10CTDBamH1R	AATGGATCCCTCAGAGTCCACACTGTCCTG
b10NTD1-68HindIIIR	TATAAGCTTGGGGGCTTTCTCAATGTCT
b10CTDBamH1F	ATAGGATCCCCAGCACACAAGCGCCACATC
b10CTDNot1R	AATTGCGGCCGCTTACTCAGAGTCCACACTGTCCTG
Irgm1V5F	ATGAAACCATCACACAGTTC
Irgm1V5R	GATCTGCGGAGGGAAGATGG
Irgm2V5F	ATGGAAGAGGCAGTTGAGTC
Irgm2V5R	AGGATGAGGAATGGAGAGTCTC
Irgm3V5F	ATGGATTTAGTCACAAAGTTG
Irgm3V5R	GTGAATTTCGGGAGGGAGGAC

### Statistical analysis

Results are represented as means ± SD. All comparisons were evaluated for statistical significance through the use of unpaired two-tailed *t* tests. When necessary, significant differences between data points were highlighted and the level of significance was depicted as: *, p<0.05; **, p<0.01; and ***, p<0.005.

## Supporting Information

Figure S1 **Ectopically expressed V5-tagged IRGM proteins localize to LDs.** Wildtype MEFs were transfected with expression plasmids for V5-tagged Irgm1, Irgm2 and Irgm3 and treated overnight with OA and IFNγ. Cells were fixed and stained with anti-V5 and BODIPY. Overlap between fluorescent anti-V5 and BODIPY staining of representative images is shown.
(TIF)

Figure S2 **Endogenous Irgm1 localizes to LDs.** Wildtype MEFs were treated overnight with OA+/−IFNγ. Cells were stained for endogenous Irgm1 and LDs using BODIPY. Whereas we detected endogenous Irgm1 on LDs, we failed to detect endogenous Irgm2 on LDs using three distinct antibodies (data not shown).
(TIF)

Figure S3 **Ectopic expression of Irgm3 in Irgm3-deficient cells dissolves aggregate staining of Irga6 in OA-treated cells.** *Irgm3* ^−/−^ and *Irgm1/m3* ^−/−^ MEFs were transduced with a retroviral expression vector for Irgm3 (pIrgm3) or an empty vector control (pBABE) and treated with OA and IFNγ. Expression of Irgm3 abolished the droplet-like staining pattern of Irga6 in both Irgm3^−/−^ and Irgm1/m3^−/−^ MEFs.
(TIF)

Figure S4 **Irgb10 protein is enriched on IRGM-deficient LDs in macrophages.** Primary bone marrow-derived marophages of the indicated genotypes were stained with BODIPY and anti-Irgb10 after overnight treatment with OA and IFNγ. Quantitative analyses of Irgb10 co-localization with LDs were performed using MBF-ImageJ software as described in Materials and Methods. Data are the representative of three independent experiments. Statistical significance of group values relative to wildtype and between marked groups is shown (***, p<0.005). Representative images are shown.
(TIF)

Figure S5 **GKS proteins mislocalize to endogenous LDs and peroxisomes in** ***Irgm1/m3*** **^−/−^.** (**A**) Irgb10 localizes to LD in the absence of OA in *Irgm1/m3* ^−/−^ MEFs. We observed additional aggregate-like, Irgb10-positive structures that did not stain with BODIPY. (**B**) A subset of these structures stained positive with MitoTracker Red. (**C**) Similar to rabbit anti-Irgb10, mouse anti-Irga6 antibody stained aggregate-like structures in *Irgm1/m3* ^−/−^ but not wildtype cells. Whereas some of these structures stained positive for BODIPY (data not shown), a subset of these structures were decorated with peroxisome marker Pmp70 stained with rabbit anti-Pmp70. (**D**) A subset of peroxisomes stained positive for mouse anti-irgm3 in wildtype cells.
(TIF)

Figure S6 **Irgb10 protein is absent from LDs in Atg5−/− MEFs.** *Atg5* ^−/−^ MEFs incubated with and without OA and IFNγ were stained for Irgb10 and LDs (BODIPY).
(TIF)

Figure S7 **An Irgb10 mutant (S82N) deficient for GTP binding fails to localize to LDs.** *Irgm1/m3* ^−/−^ MEFs were transfected with (**A**) Irgb10WT or (**B**) Irgb10^S82N^ fused to GFP. Cells were treated overnight with OA and IFNγ and stained for the LD resident protein Tip47 and DNA (Hoechst). White arrows point at inclusions. Wildtype Irgb10 but not Irgb10^S82N^ targets both inclusion and IRGM-deficient LDs. Representative images are shown.
(TIF)

Figure S8 **GBP proteins are enriched on IRGM-deficient LDs.** MEFs of the indicated genotypes were treated overnight with OA and IFNγ. Cells were stained for endogenous Gbp2, LDs (BODIPY) and DNA (Hoechst). Representative images are shown. Colocalization analyses of Gbp2 with LDs were done using MBF ImageJ software as described in Materials and Methods.
(TIF)

Figure S9 **IFNγ activation results in a decrease in LD mass as assessed by BODIPY staining in IRGM-deficient MEFs.** Wildtype and *Irgm1/m3* ^−/−^ MEFs were treated with OA to enrich for total LD mass. Cells were treated with IFNγ and stained with BODIPY. Flow cytometry was used to measure the BODIPY signal, which corresponds with LD mass. The fold change in the average mean fluorescent intensity (MFI) in response to IFNγ treatment is plotted in the panel on the right.
(TIF)
