# Supplementary figures and images for: IRG and GBP Host Resistance Factors Target Aberrant, “Non-self” Vacuoles Characterized by the Missing of “Self” IRGM Proteins
Source: PLoS Pathog. 2013 Jun 13;9(6):e1003414. doi: 10.1371/journal.ppat.1003414 (PMC3681737; doi:10.1371/journal.ppat.1003414)

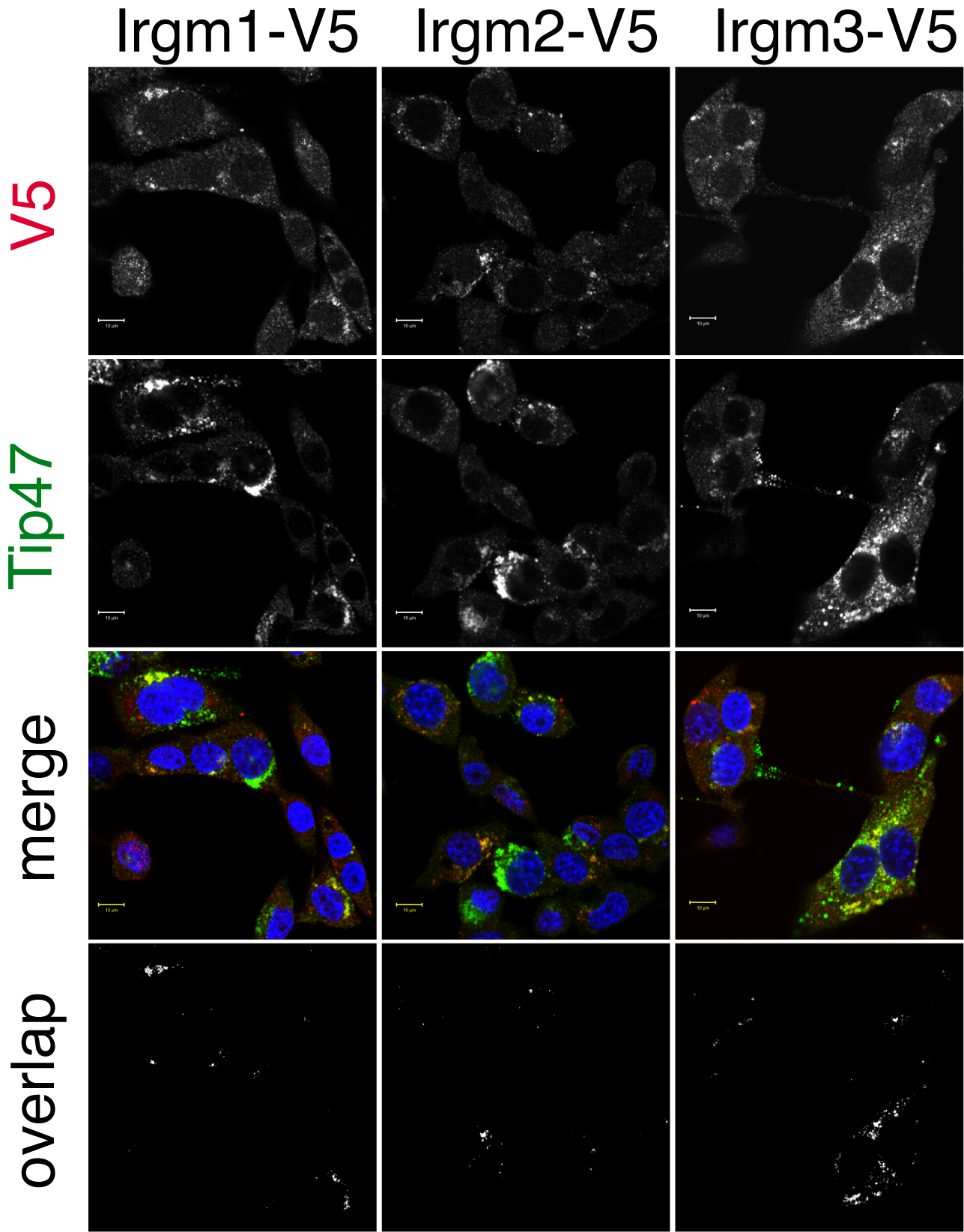

Supplement: Figure S1 — Ectopically expressed V5-tagged IRGM proteins localize to LDs. Wildtype MEFs were transfected with expression plasmids for V5-tagged Irgm1, Irgm2 and Irgm3 and treated overnight with OA and IFNγ. Cells were fixed and stained with anti-V5 and BODIPY. Overlap between fluorescent anti-V5 and BODIPY staining of representative images is shown. (TIF) [file ppat.1003414.s001.tif]

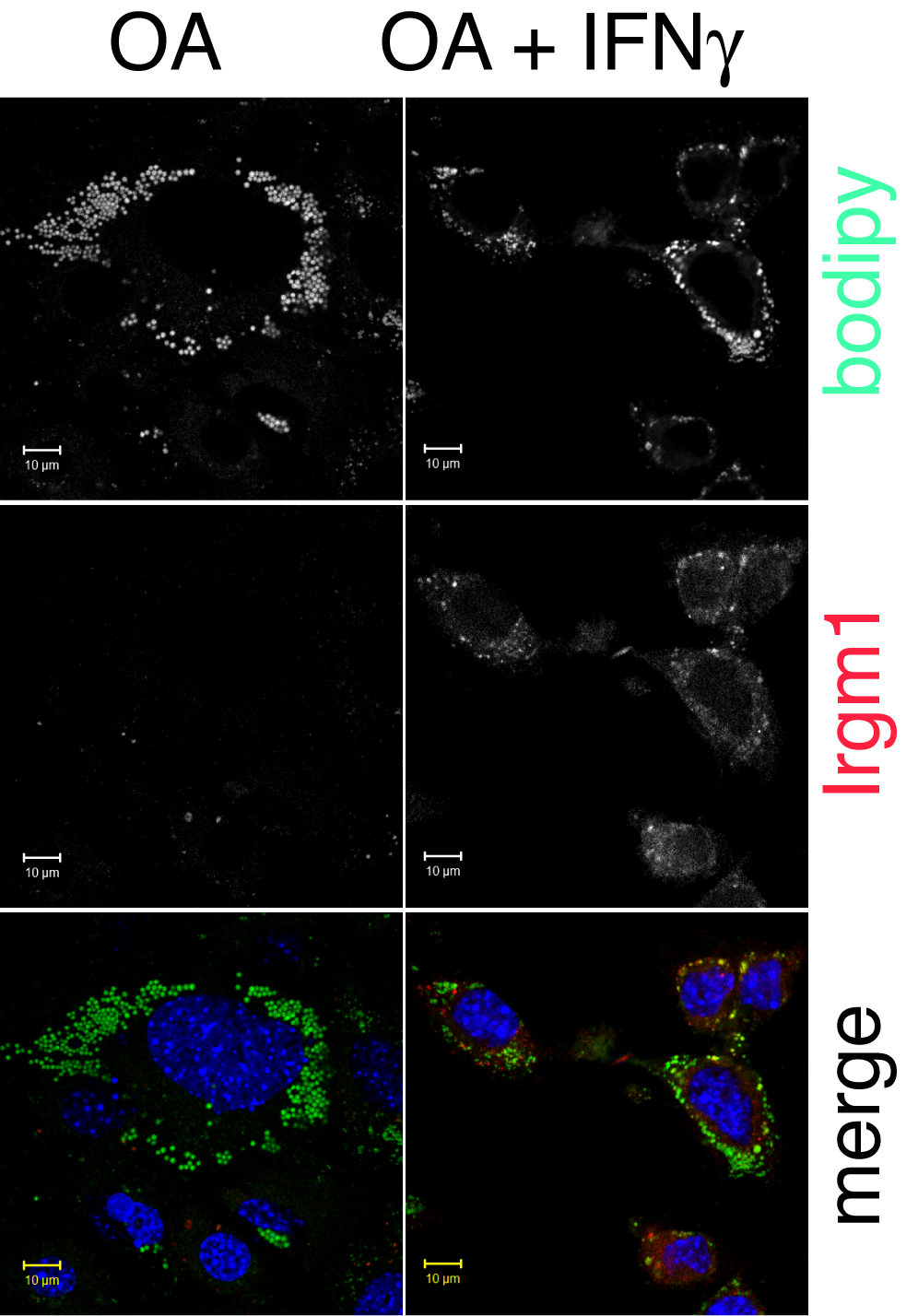

Supplement: Figure S2 — Endogenous Irgm1 localizes to LDs. Wildtype MEFs were treated overnight with OA+/−IFNγ. Cells were stained for endogenous Irgm1 and LDs using BODIPY. Whereas we detected endogenous Irgm1 on LDs, we failed to detect endogenous Irgm2 on LDs using three distinct antibodies (data not shown). (TIF) [file ppat.1003414.s002.tif]

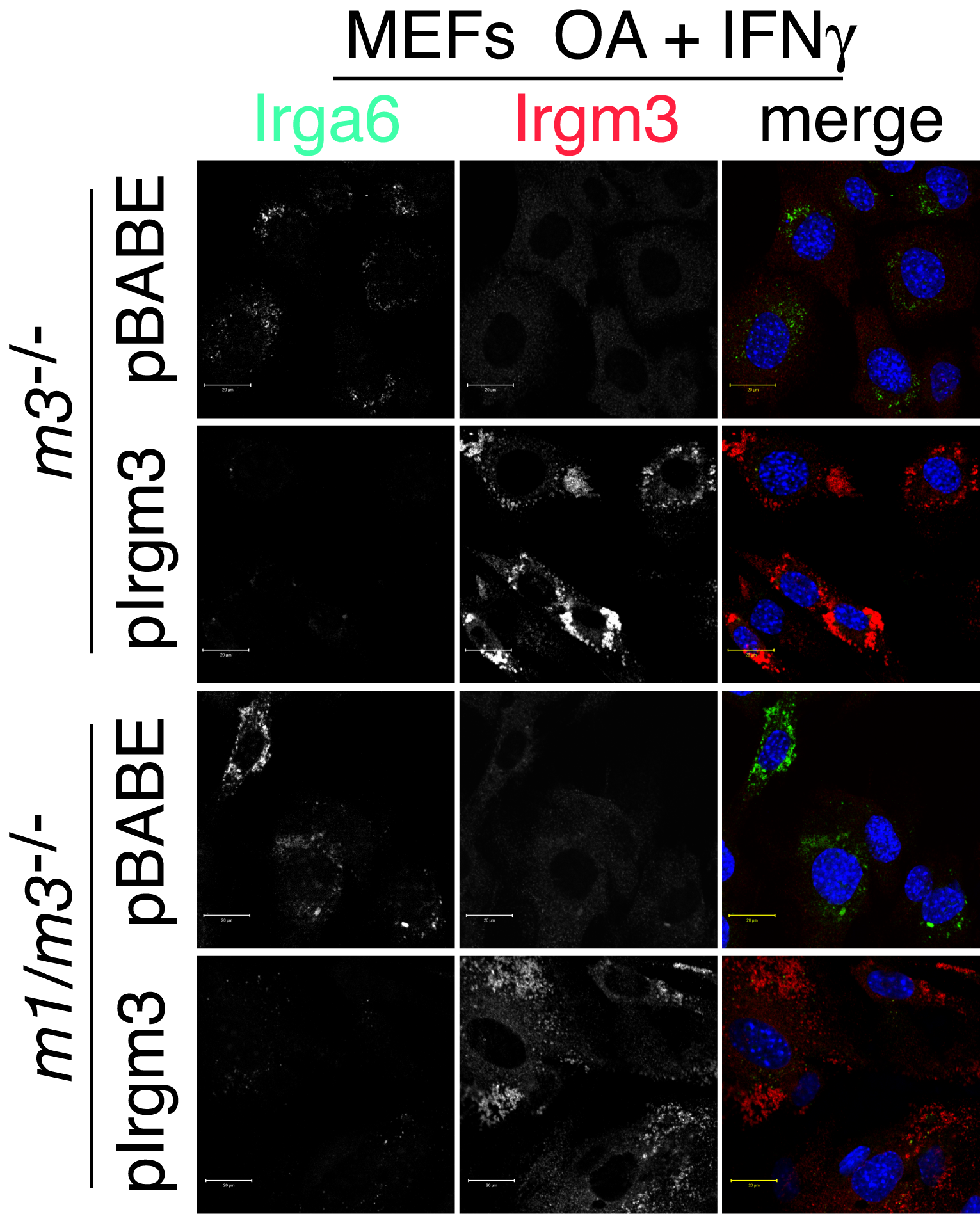

Supplement: Figure S3 — Ectopic expression of Irgm3 in Irgm3-deficient cells dissolves aggregate staining of Irga6 in OA-treated cells. Irgm3 −/− and Irgm1/m3 −/− MEFs were transduced with a retroviral expression vector for Irgm3 (pIrgm3) or an empty vector control (pBABE) and treated with OA and IFNγ. Expression of Irgm3 abolished the droplet-like staining pattern of Irga6 in both Irgm3−/− and Irgm1/m3−/− MEFs. (TIF) [file ppat.1003414.s003.tif]

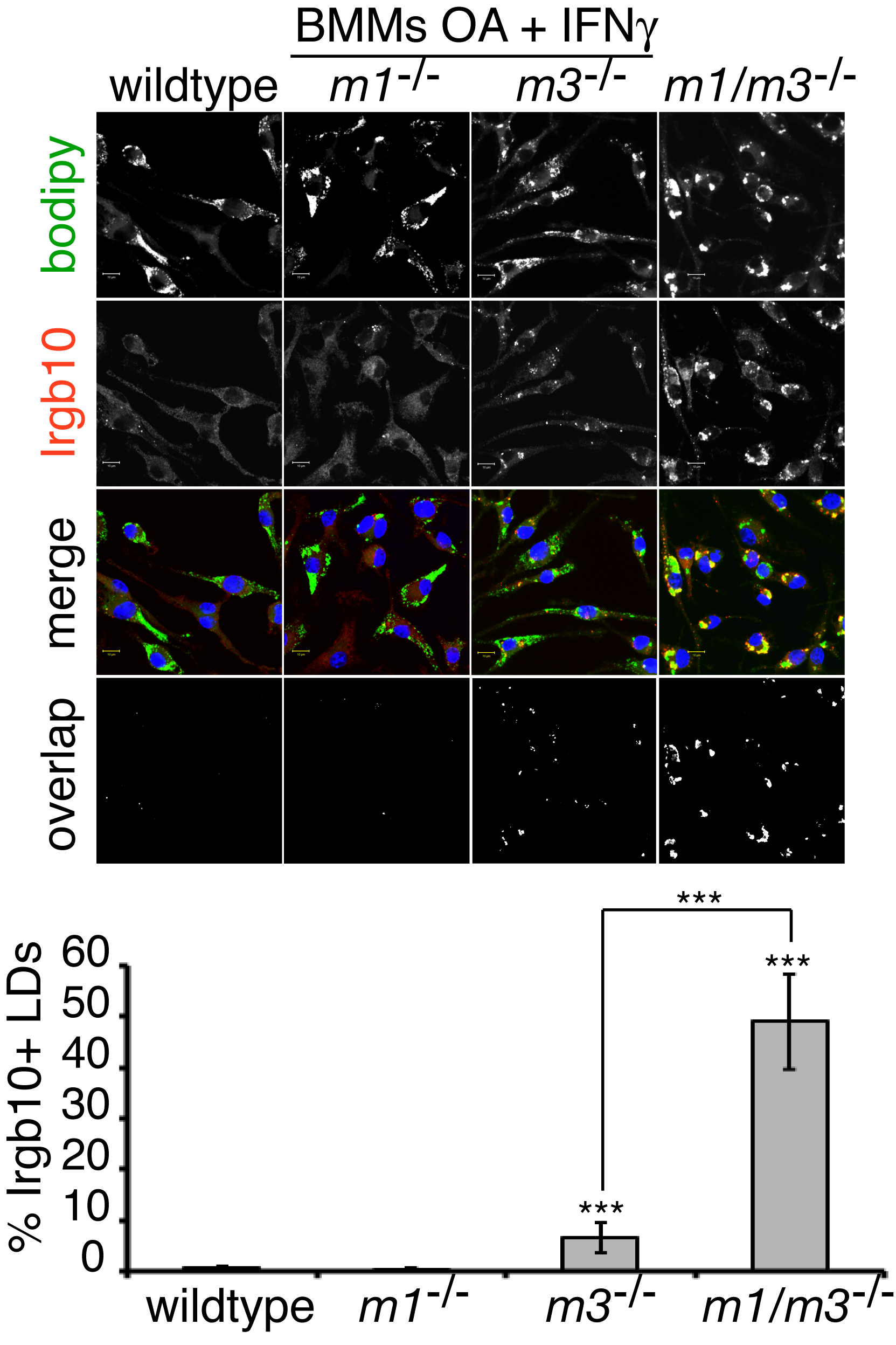

Supplement: Figure S4 — Irgb10 protein is enriched on IRGM-deficient LDs in macrophages. Primary bone marrow-derived marophages of the indicated genotypes were stained with BODIPY and anti-Irgb10 after overnight treatment with OA and IFNγ. Quantitative analyses of Irgb10 co-localization with LDs were performed using MBF-ImageJ software as described in Materials and Methods. Data are the representative of three independent experiments. Statistical significance of group values relative to wildtype and between marked groups is shown (***, p<0.005). Representative images are shown. (TIF) [file ppat.1003414.s004.tif]

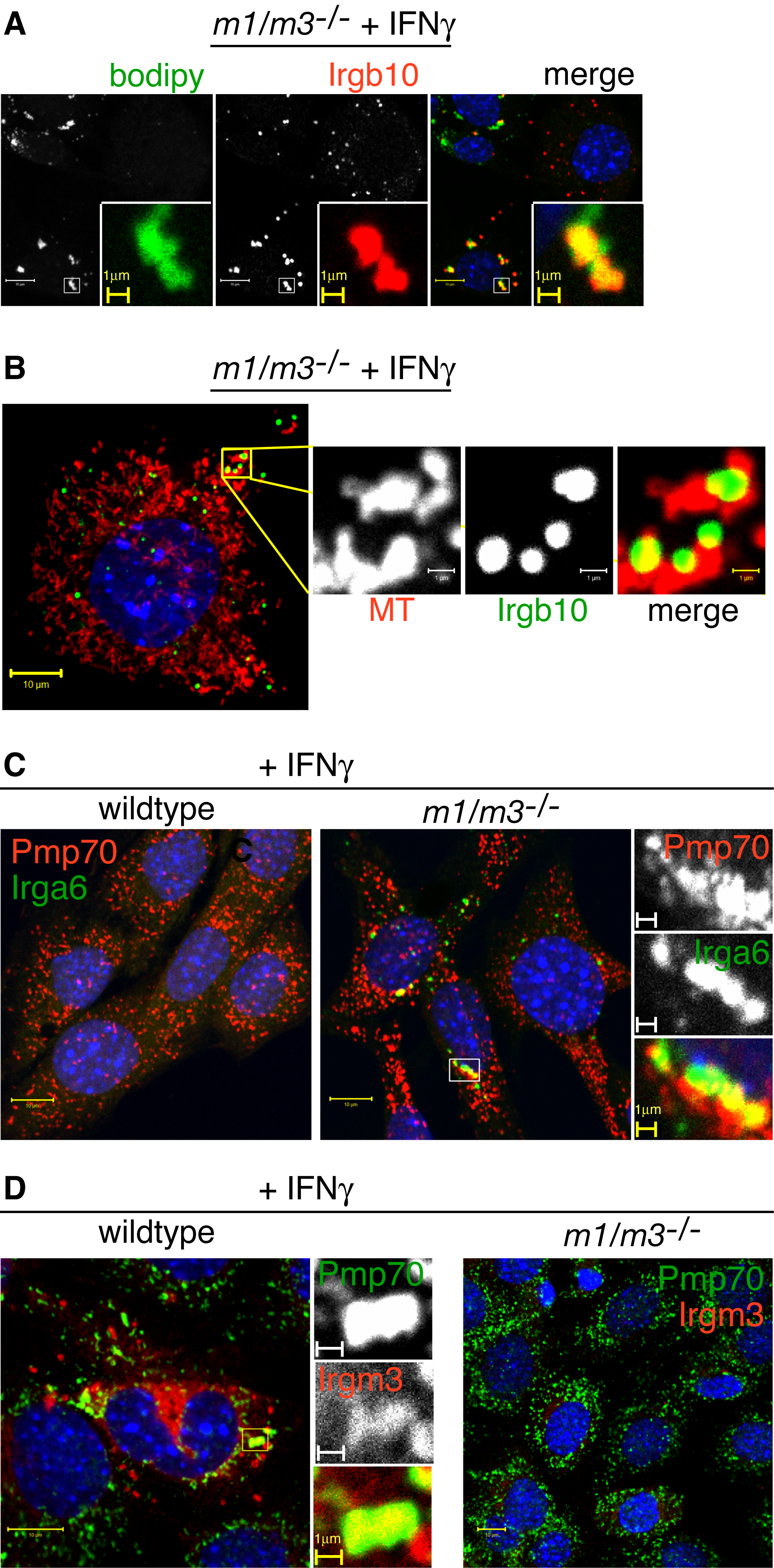

Supplement: Figure S5 — GKS proteins mislocalize to endogenous LDs and peroxisomes in Irgm1/m3 −/−. (A) Irgb10 localizes to LD in the absence of OA in Irgm1/m3 −/− MEFs. We observed additional aggregate-like, Irgb10-positive structures that did not stain with BODIPY. (B) A subset of these structures stained positive with MitoTracker Red. (C) Similar to rabbit anti-Irgb10, mouse anti-Irga6 antibody stained aggregate-like structures in Irgm1/m3 −/− but not wildtype cells. Whereas some of these structures stained positive for BODIPY (data not shown), a subset of these structures were decorated with peroxisome marker Pmp70 stained with rabbit anti-Pmp70. (D) A subset of peroxisomes stained positive for mouse anti-irgm3 in wildtype cells. (TIF) [file ppat.1003414.s005.tif]

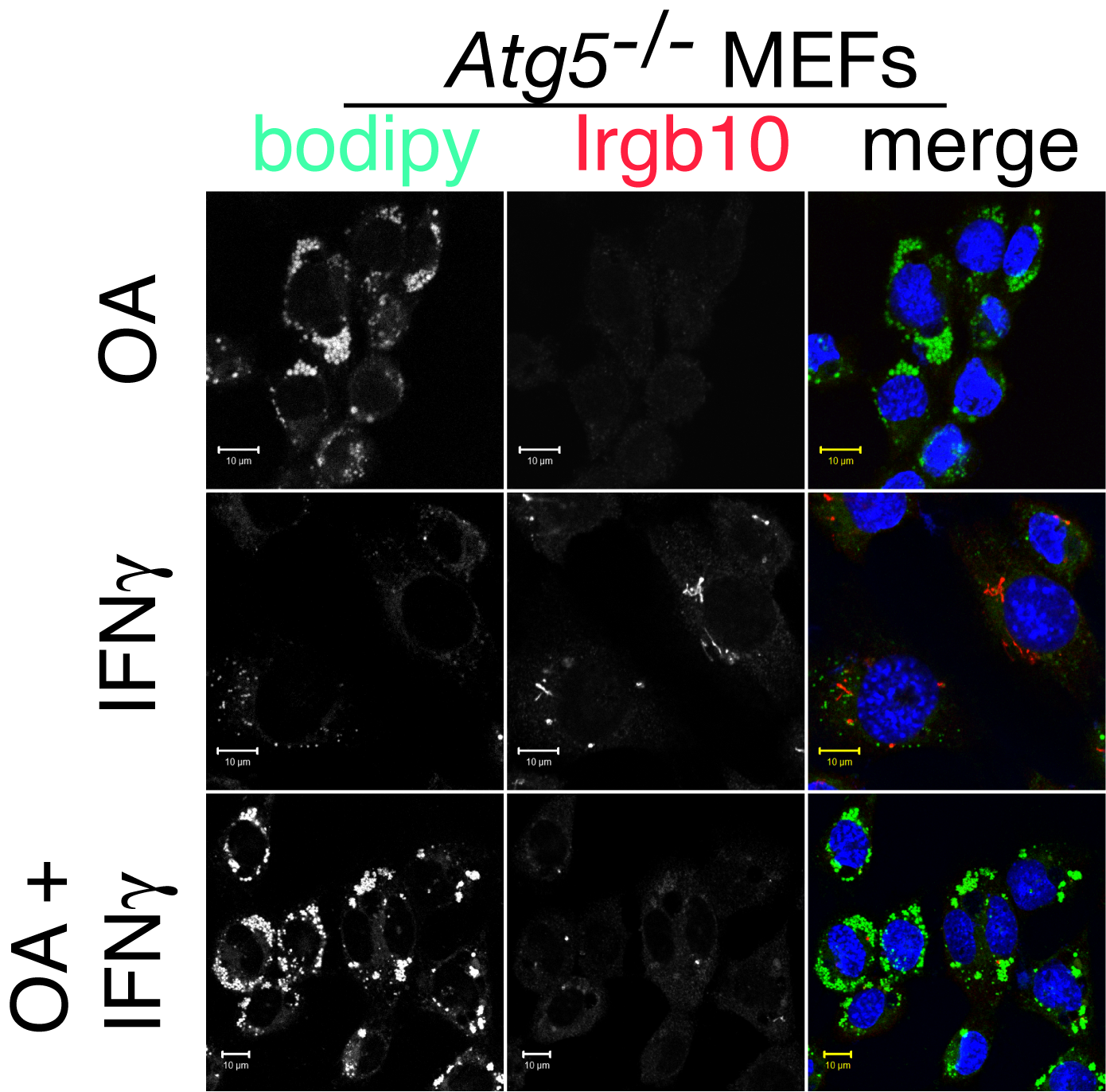

Supplement: Figure S6 — Irgb10 protein is absent from LDs in Atg5−/− MEFs. Atg5 −/− MEFs incubated with and without OA and IFNγ were stained for Irgb10 and LDs (BODIPY). (TIF) [file ppat.1003414.s006.tif]

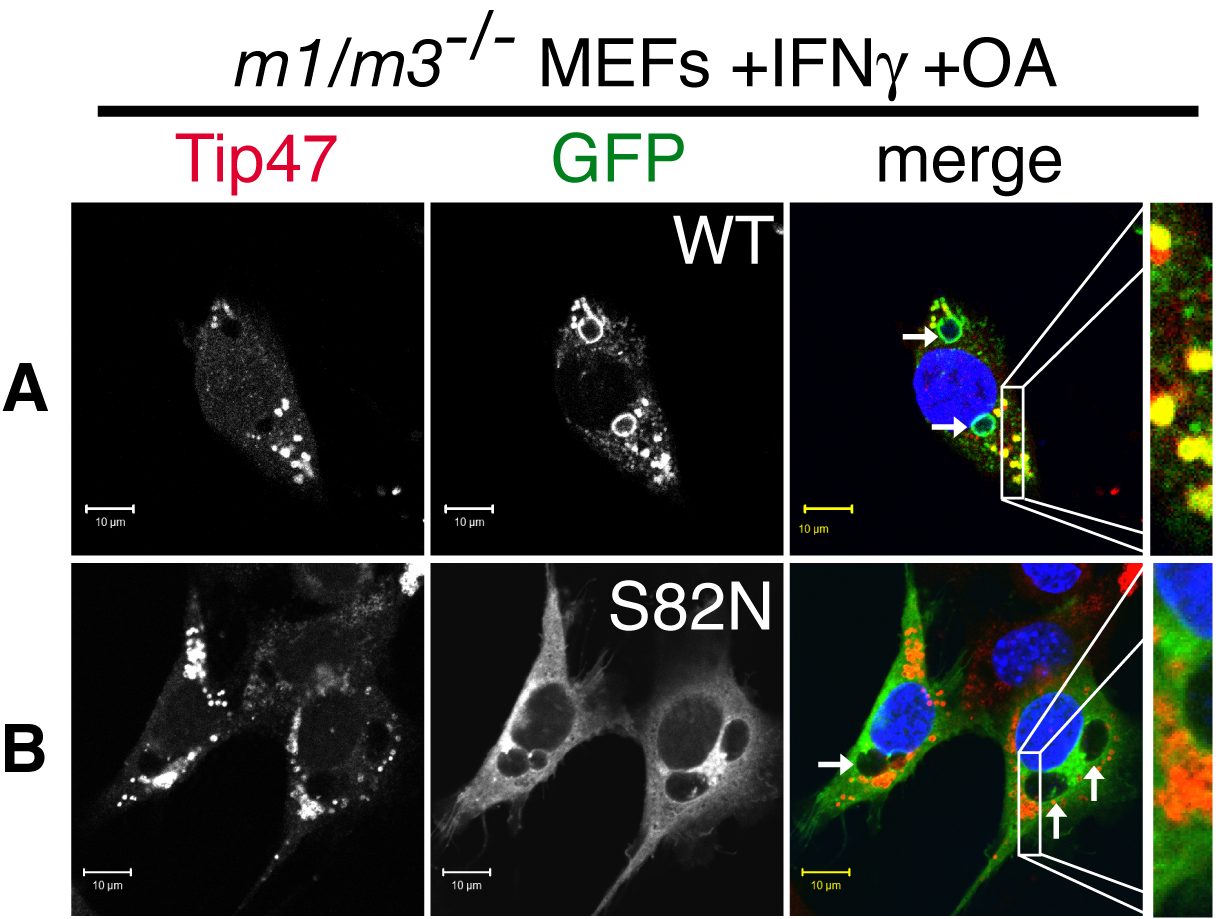

Supplement: Figure S7 — An Irgb10 mutant (S82N) deficient for GTP binding fails to localize to LDs. Irgm1/m3 −/− MEFs were transfected with (A) Irgb10WT or (B) Irgb10S82N fused to GFP. Cells were treated overnight with OA and IFNγ and stained for the LD resident protein Tip47 and DNA (Hoechst). White arrows point at inclusions. Wildtype Irgb10 but not Irgb10S82N targets both inclusion and IRGM-deficient LDs. Representative images are shown. (TIF) [file ppat.1003414.s007.tif]

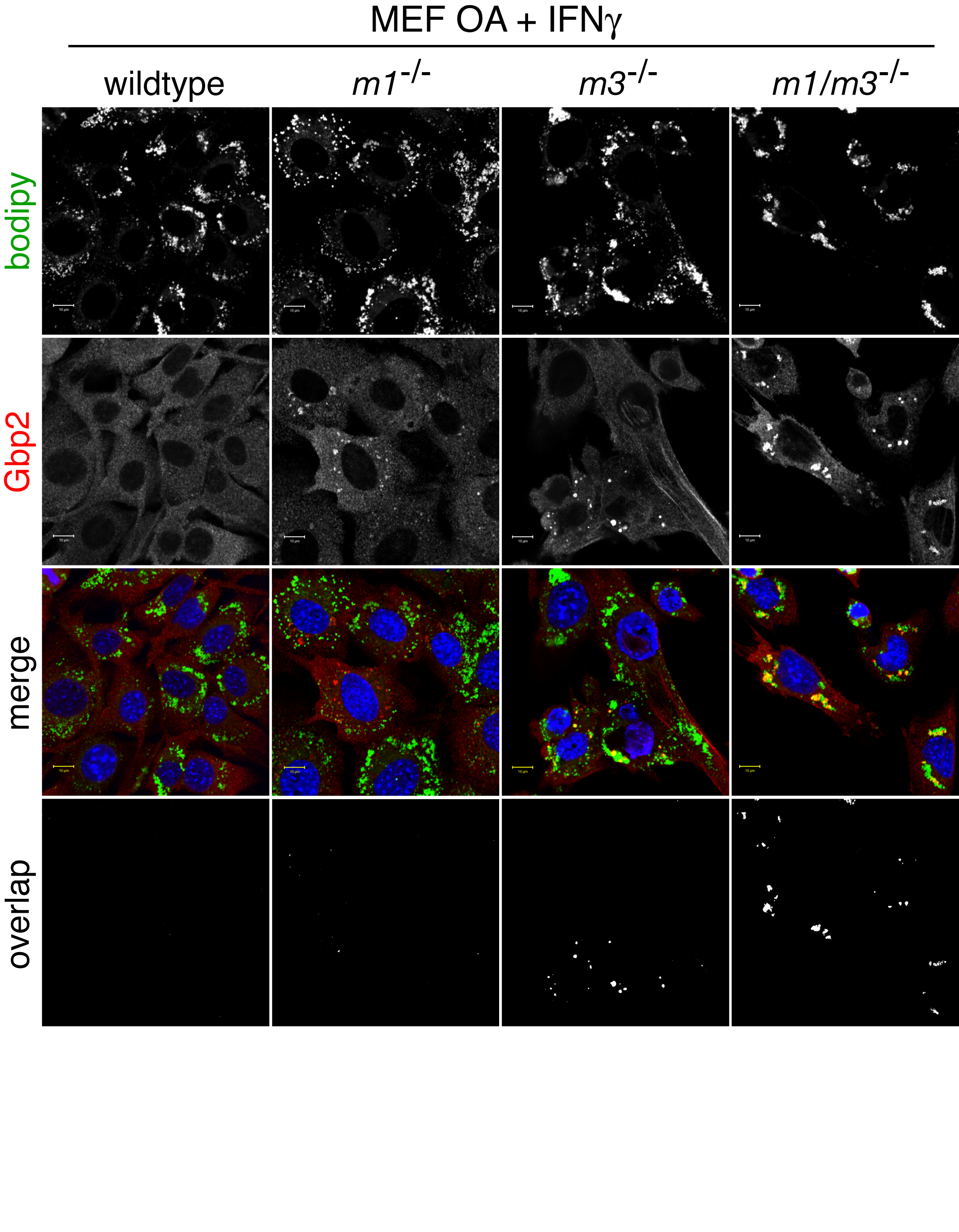

Supplement: Figure S8 — GBP proteins are enriched on IRGM-deficient LDs. MEFs of the indicated genotypes were treated overnight with OA and IFNγ. Cells were stained for endogenous Gbp2, LDs (BODIPY) and DNA (Hoechst). Representative images are shown. Colocalization analyses of Gbp2 with LDs were done using MBF ImageJ software as described in Materials and Methods. (TIF) [file ppat.1003414.s008.tif]

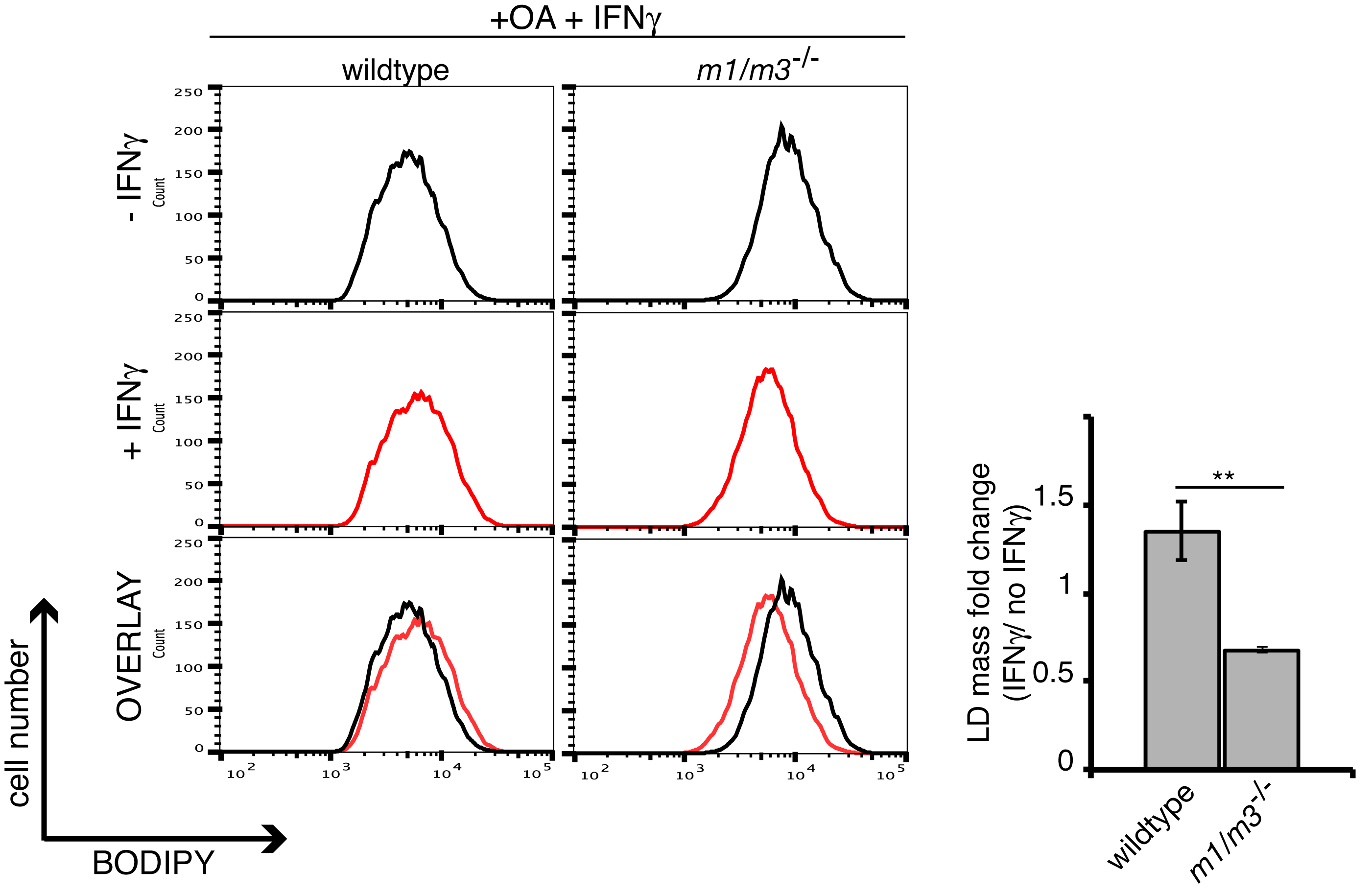

Supplement: Figure S9 — IFNγ activation results in a decrease in LD mass as assessed by BODIPY staining in IRGM-deficient MEFs. Wildtype and Irgm1/m3 −/− MEFs were treated with OA to enrich for total LD mass. Cells were treated with IFNγ and stained with BODIPY. Flow cytometry was used to measure the BODIPY signal, which corresponds with LD mass. The fold change in the average mean fluorescent intensity (MFI) in response to IFNγ treatment is plotted in the panel on the right. (TIF) [file ppat.1003414.s009.tif]
